# Biophysical mechanism of the interaction between default mode network and working memory network

**DOI:** 10.1007/s11571-021-09674-1

**Published:** 2021-04-19

**Authors:** Yue Yuan, Xiaochuan Pan, Rubin Wang

**Affiliations:** East China University of Science and Technology, Shanghai, 200237 China

**Keywords:** Default mode network, Working memory, Contained energy, Task positive/negative network, Correlation

## Abstract

Default mode network (DMN) is a functional brain network with a unique neural activity pattern that shows high activity in resting states but low activity in task states. This unique pattern has been proved to relate with higher cognitions such as learning, memory and decision-making. But neural mechanisms of interactions between the default network and the task-related network are still poorly understood. In this paper, a theoretical model of coupling the DMN and working memory network (WMN) is proposed. The WMN and DMN both consist of excitatory and inhibitory neurons connected by AMPA, NMDA, GABA synapses, and are coupled with each other only by excitatory synapses. This model is implemented to demonstrate dynamical processes in a working memory task containing encoding, maintenance and retrieval phases. Simulated results have shown that: (1) AMPA channels could produce significant synchronous oscillations in population neurons, which is beneficial to change oscillation patterns in the WMN and DMN. (2) Different NMDA conductance between the networks could generate multiple neural activity modes in the whole network, which may be an important mechanism to switch states of the networks between three different phases of working memory. (3) The number of sequentially memorized stimuli was related to the energy consumption determined by the network's internal parameters, and the DMN contributed to a more stable working memory process. (4) Finally, this model demonstrated that, in three phases of working memory, different memory phases corresponded to different functional connections between the DMN and WMN. Coupling strengths that measured these functional connections differed in terms of phase synchronization. Phase synchronization characteristics of the contained energy were consistent with the observations of negative and positive correlations between the WMN and DMN reported in referenced fMRI experiments. The results suggested that the coupled interaction between the WMN and DMN played important roles in working memory.

**Supplementary Information:**

The online version contains supplementary material available at 10.1007/s11571-021-09674-1.

## Introduction

The brain’s default mode network (DMN) was originally identified in a meta-analysis that mapped brain regions were more active in passive as compared to active tasks (often referred to as task-induced deactivation) (Buckner et al. 2008). When people are meditating, daydreaming, recalling the past, planning for the future etc., the brain is awake but at rest (also called the resting state) and the DMN is usually activated. While at task states, the DMN is usually in a low-firing mode (Raichle 2015), or so-called deactivated. Since it was first proposed in 2001, the DMN has become a core research topic in theoretical neuroscience, brain imaging, psychiatric diagnosis and medical treatment (Hu et al. 2017).

Physiological experiments showed that the low firing mode of the DMN in task states was not only an incidental phenomenon of task-state brain activity, but also played an extremely important role in maintaining normal cognitions and psychological states (Hu et al. 2017; Greicius and Menon 2004). Insufficient inhibition of the DMN in task states would reduce activity in the dorsal attention network, resulting in memory loss and cognitive impairment (Greicius and Menon 2004; Sambataro et al. 2010; Weissman 2006), which was often detected in patients with severe psychological diseases (Anticevic et al. 2012). Some functional magnetic resonance imaging (fMRI) studies have also shown that the physiological inhibition of the DMN in healthy brains was stronger than that in patients with ADHD, contributing to better accomplishment of attention tasks in healthy individuals (Uddin et al. 2008). In patients with schizophrenia and depression, the DMN was often found hyperactivated and functional hyperconnected (Whitfield-Gabrieli and Ford 2012). Behavioral research demonstrated that the hyperactivity of the DMN make it difficult for patients to transfer attention from the internal meditation to external stimuli, which induced the loss of working memory and mood control (Figueroa et al. 2017).

Due to the particular activity mode of the DMN, researchers began to explore its unique dynamics mechanism and functional significances in cognitions. Thus, many resting state network models have been put forward in order to further understand important roles of the DMN in global and cognitive brain functions (Cabral et al. 2017). Among those models, the conductance-based synaptic model (Honey et al. 2007; Honey 2009; Gollo and Breakspear 2014; Zalesky et al. 2014; Gollo et al. 1668), the Kuramoto model (Cabral et al. 2014) with gamma oscillators, and the oscillator model doped with Hopf bifurcation (Deco et al. 2017), can reproduce resting neural activity in the DMN observed in fMRI in time domain, space domain, and energy spectrum simultaneously. However, only the conductance-based synaptic model can match physiological activities observed in experimental studies better than other models, as well as overlap many theoretical task state models. The synaptic model can not only simulate the DMN’s activity in resting state but also some specific functions of dorsal attention network (Dayan 2001). However, most theoretical studies of the DMN only simulate its activity in resting state without considering the relationship between the DMN and other task networks. It is more likely that the DMN reflects its functions by interacting with other task networks. So far, there has been little understanding of the synergistic mechanism between the DMN and task networks in task states.

To simulate functions of the DMN in the task state, a task-oriented functional network is needed for cooperation. Some fMRI studies have reported that working memory is regulated by the DMN (Sambataro et al. 2010; Esposito et al. 2009; Hampson et al. 2006). Therefore, the working memory network (WMN) can be used to jointly study the dynamics of the DMN. Many experimental studies have observed couple interactions between the DMN and WMN (Gotts et al. 2020; Raichle et al. 2001). Some fMRI experiments have shown that BOLD signals in the DMN and WMN presented an inverse correlation (or called antagonism) where the DMN was inhibited (Fox et al. 2005; Dixon et al. 2016a). Nevertheless, some fMRI data indicated that the DMN and WMN were not simply antagonistic. For example, the correlation between activities in the DMN and WMN is found to be inconsistent in three phases of working memory: encoding, maintenance and retrieval (Piccoli et al. 2015). In encoding and retrieval processes, the inferior parietal lobule (IPL) in the DMN is activated and positively related with left dorsolateral prefrontal cortex (lDLPFC) and right intra-parietal sulcus (rIPS) in the WMN. While in maintenance process, the posterior cingulate/retrosplenial cortex (PCC/Rsp) and the medial prefrontal cortex (MPFC) in the DMN are inactivated and negatively correlated with the DLPFC and rIPS in the WMN (Piccoli et al. 2015). These results suggest that simple antagonistic relationship cannot fully explain the coupling mechanism between the WMN and DMN.

Since the DMN, as a large-scale dynamic neural network, involved multiple brain region activities and interactions, exploring the functional relationship between the DMN and other cognitive networks from the global perspective of brain activity could scientifically explain the role and contribution of the DMN. Previous studies had shown that neural energy theory could be effectively used on different levels of brain networks and their coupling for the construction of global neural brain models and coding (Wang and Zhu 2016; Wang et al. 2020). It has been known that theoretical energy theory could successfully interpret the neural mechanism of brain hemodynamic phenomena (Peng et al. 2021). Why the amount of data in biological visual system would significantly reduce but not affect the external world visual cognition could also be explained by energy theory (Zhong and Wang 2020). Quantitative neural energy analysis of spatial cognition and maximum information coding of various animals also obtained results that well matched the experimental data (Wang et al. 2018, 2019).

In order to understand the coupling mechanism between the WMN and DMN, a network model has been proposed and implemented to simulate a working memory process containing encoding, maintenance and retrieval phases in this article. Using this model, we explored that the change of excitatory neurotransmitter conductance between the WMN and DMN would cause significant diversification in firing patterns of the entire network. The use of neural energy theory could not only reproduce energy consumption changes in the dynamic interaction of DMN and WMN, but also present the quantitative relationship between firing rate and energy consumption. Phase locking analysis on the contained energy of population neurons demonstrated activity correlations in the DMN and WMN. We found negative or positive correlations between the WMN and DMN during different phases in a complete working memory process, which was consistent with observations reported in a fMRI study (Piccoli et al. 2015). The result could be utilized to make relevance between negative and positive correlations of WMN and DMN.

## Methods

### Theoretical model

Based on the DMN characteristic, being deactivated in task state and highly activated in resting state, the model proposed in this paper consists of a Task Positive Network (TPN, representing the WMN) and a Task Negative Network (TNN, representing a sub-network of the DMN) to simulate coupled interactions between the DMN and WMN (Fox et al. 2005; Dixon et al. 2016a; Cheng et al. 2020; Andreou et al. 2018). The TPN has low firing rates in the resting state and high firing rates in the task state so that it corresponds to the WMN. The TNN fires high in resting state and low in task state so that it corresponded to the DMN.

**Fig. 1:**
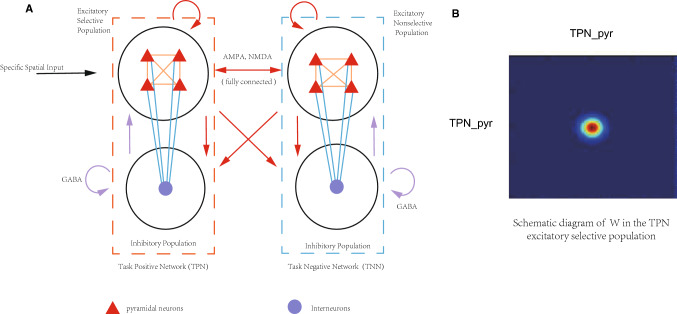
**a** The framework of TPN–TNN model. Red triangles represent pyramidal cells, while violet dots represent interneurons. The orange dashed box on the left represents the TPN, which physiologically represents the dorsal lateral prefrontal cortex (DLPFC). The blue dashed box on the right represents the TNN, which physiologically represents the posterior cingulate cortex (PCC). Arrows indicate directions of neurotransmitter transmission between neuron populations. Red arrows indicate excitatory synapses of AMPA and NMDA, while violet arrows indicate inhibitory synapses of GABA; and the black arrow indicates input stimulation. **b** Two-dimensional representation of the connection weights in TPN pyramidal cells population (TPN_pyr), showing a preference direction of 180°

In this model, each network contained 2048 excitatory neurons (pyramidal cells) and 512 inhibitory neurons (interneurons). The ratio of excitatory and inhibitory neurons was 4:1 (Markram et al. 2004; Sieghart 2012; Sillito 1975; Priebe and Ferster 2008; Ozeki et al. 2009; Liu et al. 2010). Only pyramidal cells in the TPN have direction selectivity, while other neurons didn’t show direction selectivity either in the TPN or in the TNN (Hollup et al. 2001). The connections between the TPN and TNN were set up by NMDA and AMPA channels, and the neuronal projections within each network were constructed by NMDA, AMPA and GABA channels according to excitatory or inhibitory synapses. Figure 1 shows the schematic architecture of the model (Fig. 1a) and direction selective weights of excitatory neurons in the TPN (Fig. 1b).

In the TPN–TNN coupled network model (Fig. 1a), the activity of each neuron was calculated using Integrate-and-Fire model (I–F model) with synaptic gated modulation (Compte 2000; Wei et al. 2012). The membrane potentials and synaptic currents of the excitatory and inhibitory neurons in the TPN and TNN were described in Eqs. 1–8, respectively.

1 $$ C_{m\_pyr} \frac{{dV_{TPNpyr} (t)}}{dt} = - g_{L\_pyr} (V_{TPNpyr} (t) - V_{L} ) - I_{TPNpyr\_syn} $$

2 $$ C_{m\_pyr} \frac{{dV_{TNNpyr} (t)}}{dt} = - g_{L\_pyr} (V_{TNNpyr} (t) - V_{L} ) - I_{TNNpyr\_syn} $$

3 $$ \begin{aligned} I_{TPNpyr\_syn} (t) & = I_{TPNpyr\_AMPA} + I_{TPNpyr\_NMDA} + I_{TPNinh\_GABA} \\ & \quad + I_{TNNpyrtoTPNpyr\_AMPA} + I_{TNNpyrtoTPNpyr\_NMDA} + I_{noise} + I_{ext} \\ \end{aligned} $$

4 $$ \begin{aligned} I_{TNNpyr\_syn} (t) & = I_{TNNpyr\_AMPA} + I_{TNNpyr\_NMDA} + I_{TNNinh\_GABA} \\ & \quad + I_{TPNpyrtoTNNpyr\_AMPA} + I_{TPNpyrtoTNNpyr\_NMDA} + I_{noise} \\ \end{aligned} $$

5 $$ C_{m\_inh} \frac{{dV_{TPNinh} (t)}}{dt} = - g_{L\_inh} (V_{TPNinh} (t) - V_{L} ) - I_{TPNinh\_syn} $$

6 $$ C_{m\_inh} \frac{{dV_{TNNinh} (t)}}{dt} = - g_{L\_inh} (V_{TNNinh} (t) - V_{L} ) - I_{TNNinh\_syn} $$

7 $$ \begin{aligned} I_{TPNinh\_syn} (t) & = I_{TPNinh\_GABA} + I_{TPNpyrtoTPNinh\_AMPA} + I_{TPNpyrtoTPNinh\_NMDA} \\ & \quad + I_{TNNpyrtoTPNinh\_AMPA} + I_{TNNpyrtoTPNinh\_NMDA} + I_{noise} \\ \end{aligned} $$

8 $$ \begin{aligned} I_{TNNinh\_syn} (t) & = I_{TNNinh\_GABA} + I_{TPNpyrtoTNNinh\_AMPA} + I_{TPNpyrtoTNNinh\_NMDA} \\ & \quad + I_{TNNpyrtoTNNinh\_AMPA} + I_{TNNpyrtoTNNinh\_NMDA} + I_{noise} \\ \end{aligned} $$

Equations 1, 2 described the membrane potential change of one pyramidal cell in the TPN and TNN, respectively. Equations 5, 6 were applied to calculate the membrane potential of one inhibitory neuron in the TPN and TNN. C_m_ was membrane capacitor, g_L_ was leakage conductance, V_L_ was resting potential, V(t) was membrane potential, and I_syn_ was the total synaptic current into neurons. Different neuron population and networks were distinguished by their subscripts.

Synaptic currents were divided into AMPA, NMDA and GABA currents (Eqs. 3, 4, 7, 8). Both currents within each network and between the TPN and TNN were calculated basically with the same method (except for terms with subscript TPN_pyr). AMPA and NMDA currents were given by excitatory neurons, while GABA currents were given by inhibitory neurons. Specific calculation methods have been shown as following equations (Eqs. 9–15). Values of constant parameters used in the simulation could be found in the supplementary material.

9 $$ I_{i,NMDA} = (V_{i} - V_{E} )\sum\limits_{j} {\frac{{g_{ji,NMDA} S_{j,NMDA} }}{{1 + [{\text{Mg}}^{2 + } ]\exp ( - 0.062V_{i} /3.57)}}} $$

10 $$ I_{i,AMPA} = (V_{i} - V_{E} )\sum\limits_{j} {g_{ji,AMPA} S_{j,AMPA} } $$

11 $$ I_{i,GABA} = (V_{i} - V_{I} )\sum\limits_{j} {g_{ji,GABA} S_{j,GABA} } $$

12 $$ \frac{{dS_{j,AMPA} }}{dt} = - \frac{{S_{j,AMPA} }}{{\tau_{AMPA} }} + \sum\limits_{k} {\delta (t - t_{k} )} $$

13 $$ \frac{{dS_{j,GABA} }}{dt} = - \frac{{S_{j,GABA} }}{{\tau_{GABA} }} + \sum\limits_{k} {\delta (t - t_{k} )} $$

14 $$ \frac{dx}{{dt}} = - \frac{x}{{\tau_{NMDA,rise} }} + \sum\limits_{k} {\delta (t - t_{k} )} $$

15 $$ \frac{{dS_{j,NMDA} }}{dt} = - \frac{{S_{j,NMDA} }}{{\tau_{NMDA,decay} }} + \beta x(1 - S_{j.NMDA} ) $$

In Eqs. 9–15, i represented the label number of neurons other than TPN excitatory neurons. Different g indicated synaptic conductance of different neurotransmitter receptors, distinguished by its subscripts. τ with different subscripts were time constants of various synaptic gate-controlled variables. S and x were intermediate variables for differentiate calculation. Summation symbol represented summing the action potentials of the presynaptic neuron j at timepoint t_k_.

Excitatory neurons (TPN_pyr) in the TPN received an external input with a preferred direction (Hollup et al. 2001) which presented a normal distribution centered on the preferred direction angle (Eq. 21). Since pyramidal cells in the TPN were selective to directional stimuli, their connection weights were formed by a normal distribution centered on the particular pyramidal cell with the preferred direction $\theta_{i}$ (Eqs. 19, 20, Fig. 1b). This contribution determined different NMDA currents among pyramidal cells in the TPN (Eq. 16). In the TPN, AMPA currents and GABA currents were also calculated by Eqs. 10, 13, but NMDA currents of excitatory neurons were calculated by the following Eqs. 16–20:

16 $$ I_{TPNpyr\_i,NMDA} = (V_{TPNpyr\_i} - V_{E} )\sum\limits_{TPNpyr\_j} {\frac{{g_{TPNpyr\_ji,NMDA} S_{TPNpyr\_j,NMDA} W(\theta_{TPNpyr\_i} - \theta_{TPNpyr\_j} )}}{{1 + [Mg^{2 + } ]\exp ( - 0.062V_{TPNpyr\_i} /3.57)}}} $$

17 $$ \frac{{dx_{TPNpyr} }}{dt} = - \frac{{x_{TPNpyr} }}{{\tau_{NMDA,rise} }} + \sum\limits_{k} {\delta (t - t_{k} )} $$

18 $$ \frac{{dS_{TPNpyr\_j,NMDA} }}{dt} = - \frac{{S_{TPNpyr\_j,NMDA} }}{{\tau_{NMDA,decay} }} + \beta x_{TPNpyr} (1 - S_{TPNpyr\_j.NMDA} ) $$

19 $$ W\left( {\theta_{TPNpyr\_i} - \theta_{TPNpyr\_j} } \right) = J^{ - } + \left( {J^{ + } - J^{ - } } \right)\exp \left( { - \frac{{\left( {\theta_{TPNpyr\_i} - \theta_{TPNpyr\_j} } \right)^{2} }}{{2\sigma^{2} }}} \right) $$

20 $$ \frac{1}{360}\int_{0}^{360} {W\left( {\theta_{TPNpyr\_i} - \theta_{TPNpyr\_j} } \right)} d\theta_{TPNpyr\_j} = 1 $$

The external input stimulus (Eq. 21) and the background activity (Eq. 22) were calculated as follows. The AMPA gated variable in the background response (Eq. 22) was calculated in the same way as Eq. 12. $\theta_{in,\alpha }$ represented preferred direction as same as $\theta_{i}$. $I_{0}$ represented the maximum stimuli current. $\sigma_{{\text{s}}}$ was a constant. Specific parameter values have been listed in the supplementary material.

21 $$ I_{ext} (\theta ) = \sum\limits_{\alpha = 1}^{n} {\frac{{I_{0} }}{{\sqrt {2\pi } \sigma_{s} }}} \exp \left[ { - \frac{{\left( {\theta - \theta_{in,\alpha } } \right)^{2} }}{{\sigma_{s} }}} \right] $$

22 $$ I_{i,noise} = (V_{i} - V_{E} )\sum\limits_{j} {g_{noise,AMPA} S_{noise,AMPA} } $$

It is worth noting that, due to the oscillatory activity caused by AMPA channels, AMPA conductance between neurons (Eqs. 10, 12) could sometimes be reduced to negligible in theoretical simulation in order to highlight stable anticorrelation results between the TPN and TNN (Cheng et al. 2020). However, some studies have shown that the oscillation caused by AMPA was an important factor in neuronal rhythmic activities (Fuchs et al. 2001; Zhang et al. 2019), closely related to formation and retrieval of memories (Rogawski 2013). Moreover, the rapid firing caused by AMPA was a possible causation of synaptic plasticity (Carver et al. 2008). Therefore, we accepted the oscillation caused by AMPA and kept the normal AMPA impact on the whole network, so as to find out the possible switching mechanisms from one phase to another phase in the working memory process, and to find out a more reliable coupling mechanism between the DMN and WMN. In this paper, the AMPA conductance in the network was adjusted to the similar magnitude of NMDA conductance, and the ratio of AMPA/NMDA was modulated to make the oscillation less noisily. Detailed model parameters have been listed in supplementary material. Most parameters were consistent with physiological synaptic parameters referred to Compte et al. (2000) and Wei et al. (2012). Simulation results illustrated in the paper were only firing patterns of excitatory neurons.

**Fig. 2:**
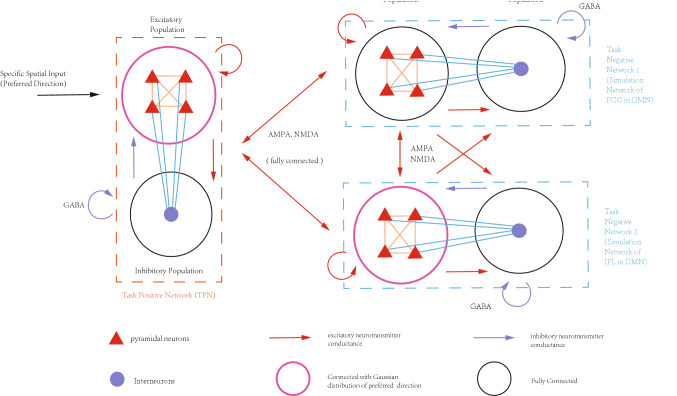
The WMN–DMN coupling structure composed of one TPN and two TNNs. Magenta circles represent neuron populations with normal distribution of preferred directions to assign weights. Black circles represent neuron populations without the normal distribution. Connections between TPN and two TNNs are simplified, which were similarly shown in Fig. 1a

The network model shown in Fig. 1a has one TPN (representing the WMN) and one TNN (representing a subnetwork in the DMN). Some experimental studies demonstrated that the DLPFC, the major part of the WMN, coupled with different brain regions of the DMN in different phases of working memory, and the DLPFC showed positive or negative correlations with those different default network brain regions (Piccoli et al. 2015). The important question was that how the WMN interplayed with different sub-networks in the DMN to generate positive or negative correlations in different phases in a working memory process. In order to investigate this issue, we had to extend the two-network model as shown in Fig. 1a to a model consisting of three networks. One TPN represented the WMN and two mutually coupled TNNs indicated two sub-networks (e.g. PCC and IPL) of the DMN (Fig. 2). We still used the basic framework of the TPN–TNN model, but the DMN was divided into 2 mutually coupled TNNs to study a complete working memory process. The specific network architecture has been shown in Fig. 2.

The TNN1 at the upper right of Fig. 2 represented PCC showing the negative correlation with the WMN during the maintenance phase of working memory. The TNN2 at the lower right of Fig. 2 represented IPL showing positive correlation with the WMN during the encoding and retrieval phases of working memory. The weights within the excitatory neuron population in TNN2 have directional selectivity (Eqs. 16–20). The excitatory neurons in TNN1 are fully connected each other without directional selectivity (Fig. 2). The synaptic currents of pyramidal cells in the TPN and two TNNs were calculated by Eqs. 23–25, similar to the Eq. 3, 4 except that it was the interaction among three networks. Synaptic currents, membrane potentials, external stimulus and background response were calculated in the same way by Eqs. 9–22 shown above.

23 $$ \begin{aligned} I_{TPN\_syn} (t) & = I_{TPNpyr\_AMPA} + I_{TPNpyr\_NMDA} + I_{TPNinh\_GABA} \\ & \quad + I_{TNN1toTPNpyr\_AMPA} + I_{TNN1toTPNpyr\_NMDA} + I_{TNN2toTPNpyr\_AMPA} \\ & \quad + I_{TNN2toTPNpyr\_NMDA} + I_{noise} + I_{ext} \\ \end{aligned} $$

24 $$ \begin{aligned} I_{TNN1\_syn} (t) & = I_{TNN1pyr\_AMPA} + I_{TNN1pyr\_NMDA} + I_{TNN1inh\_GABA} \\ & \quad + I_{TPNtoTNN1pyr\_AMPA} + I_{TPNtoTNN1pyr\_NMDA} + I_{TNN2toTNN1pyr\_AMPA} \\ & \quad + I_{TNN2toTNN1pyr\_NMDA} + I_{noise} \\ \end{aligned} $$

25 $$ \begin{aligned} I_{TNN2\_syn} (t) & = I_{TNN2pyr\_AMPA} + I_{TNN2pyr\_NMDA} + I_{TNN2inh\_GABA} \\ & \quad + I_{TPNtoTNN2pyr\_AMPA} + I_{TPNtoTNN2pyr\_NMDA} { + }I_{TNN1toTNN2pyr\_AMPA} \\ & \quad + I_{TNN1toTNN2pyr\_NMDA} + I_{noise} \\ \end{aligned} $$

The three-phase transition of working memory was realized by modifying NMDA channels connecting from the TPN to the TNN1 or to the TNN2 at certain timepoints from maintenance to retrieval phase. The stimulation phase period was 750–1000 ms. The encoding phase period was defined as 750–1750 ms, a little longer than the stimulation period. The maintenance phase period was defined as 1750–8000 ms in which no external stimuli were inputted and the activity in the networks kept in stable states. The retrieval phase period was defined as 8000–9000 ms in which stored information was retrieved to be used to complete the working memory task. Those time windows referred to the experimental task arrangement of Piccoli (2015) where the encoding phase (with stimuli) was 2000 ms, maintenance phase was 7000–10,000 ms, retrieval phase was 1000 ms (Piccoli et al. 2015). Combining the reference with the theoretical results of TPN with single TNN, encoding and maintenance periods were shortened in this study. See the supplementary materials for specific simulation parameters.

### Contained energy

The excitatory neurotransmitter AMPA mentioned in “Theoretical method” section would cause a strong oscillation in the model. Because the emergence of synchronous oscillation in high frequency would make firing rate data very noisy, firing rate curves could not well reflect positive correlation or negative correlation between the TPN and TNN. Therefore, another parameter needed to be introduced to more intuitively represent neuronal population activities.

Based on the research of Moujahid on the energy of HH neurons (Moujahid et al. 2011; Wang et al. 2017), we proposed a method to calculate the energy of I-F neurons with synaptic gating modulation.

First, take a HH neuron as an example. In each simulation time unit dt, the electric energy contained in one HH neuron is:

26 $$ H(t) = \frac{1}{2}CV^{2} + H_{Na} + H_{K} + H_{l} $$

The first term in Eq. 26 is the electrical energy accumulated by the membrane capacitance, while the last three terms seem the energy of sodium and potassium pump as the internal storage energy of a chemical battery. According to the I–F neuron model and the basic form of synaptic mechanism given in Eqs. 1, 2, it can be inferred that the membrane potential *V*(*t*) has included the neurotransmitter regulation when updating each time interval dt. Thus, for the I-F neuron model (see Eqs. 1,2), the electric energy contained in a single I–F neuron in each simulation time unit dt can be obtained as follows:

27 $$ H_{i} (t) = \frac{1}{2}C_{m} V(t)^{2} + H_{l} $$

Here, i in Eq. 27 represented the index of a neuron in the population. The leakage energy H_l_ can be obtained from the leakage voltage term in Eq. 1. So, the electrical energy contained in each population with N neurons (the black and magenta circles in Figs. 1a and 2) is:

28 $$ H(t) = \sum\limits_{i = 1}^{N} {H_{i} (t)} $$

It should be noted that in general physics, the so-called power is the energy consumption in per unit time. It can be obtained by differentiating H(t) on time with addition of electrochemical energy consumption, and is different from "contained energy" in this article. The contained energy here means the total electrical energy accumulated in a single I–F neuron.

**Fig. 3:**
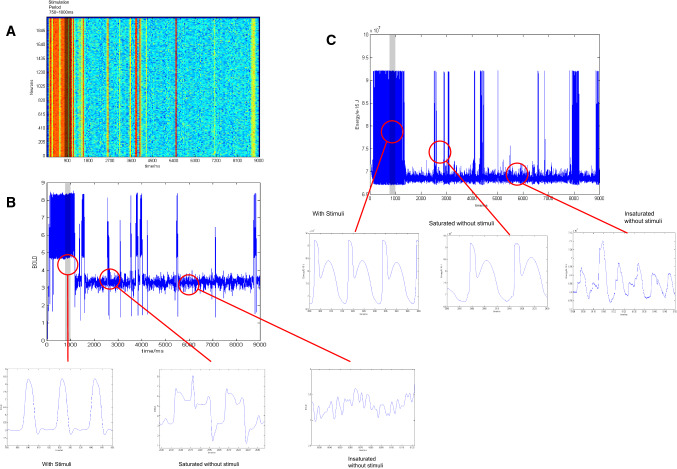
The comparison of TNN firing rate, simulated BOLD signal, and contained energy. The gray bars represented the stimulation time period (750–1000 ms). **a** The firing rate result of TNN; **b** BOLD signal calculated from firing rates of TNN excitatory neurons by using hemodynamic algorithm (Friston 2003; Faro et al. 2010); **c** Contained energy in the TNN. The amplified oscillation of BOLD signal and contained energy marked with red circle was shown in sequences from left to right in (**b**) and (**c**). Left: when the stimulus was applied; Middle: when the stimulus was removed but the oscillation was strong; Right: when the stimulus was removed but the oscillation was weak

The contained energy could better link the results of theoretical models with BOLD signals measured in fMRI experiments. Using the algorithm proposed by Friston to convert neural activity into BOLD signals (Friston 2003; Faro et al. 2010), firing rates of the TNN excitatory neurons in a trial were converted into a BOLD-like signal here. The simulated BOLD signal was compared with firing rates and the contained energy from the same simulation trial (Fig. 3).

It could be observed that the total energy of all excitatory neurons in the TNN was nearly consistent with the firing rate curve and the simulated BOLD signal based on firing rates. Even better, the contained energy (Fig. 3c) could reflect special oscillation waveforms in the TNN during and after the stimulation period, while firing rates appeared noisy and the BOLD signal was unable to display a characteristic waveform. This phenomenon might be caused by the insufficient TNN inhibition or the abnormal increase of firing rates due to the oscillation caused by AMPA. However, some researches had shown that such waveforms in the membrane potential could reflect directional selectivity (Carver et al. 2008). The contained energy calculated from the membrane potential displayed more characteristics of directional selectivity than the membrane potential itself in the TPN.

Compared with the firing rate and the simulated BOLD signal, the contained energy is more informative and has a more concise definition, which makes it suitable for analyzing properties of population activities.

### Phase locking value (PLV) and phase lag index (PLI)

The contained energy in the TNN and TNN showed a special oscillation pattern at the energy level (Fig. 3), so an index that reflected oscillatory relations between two rhythmic signals was needed to identify the relationship between the TPN and TNN in different working memory phases. In this article, Phase Locking Value (PLI) (Aydore et al. 2013) and Phase Lag Index (PLI) (Stam et al. 2007) were used to analyze phase synchronization of energy oscillations between the TPN and TNN1, between the TPN and TNN2 separately in three stages of working memory. It provided a possible method to verify that the negative and positive correlations between the WMN and DMN could be integrated and transformed.

Considering a pair of signals S_1_(t) and S_2_(t) with similar frequencies, their analytic signals can be obtained by Hilbert transform (HT):

29 $$ z_{i} (t) = s_{i} (t) + jHT(s_{i} (t))\quad (i = \{ 1,2\} ,j = \sqrt { - 1} ) $$

In this way, the relative phases of the two analytic signals can be calculated by the following equation:

30 $$ \Delta \phi (t) = \arg \left( {\frac{{z_{1} (t)z_{2}^{*} (t)}}{{\left| {z_{1} (t)} \right|\left| {z_{2} (t)} \right|}}} \right) $$

Instantaneous PLV is defined as:

31 $$ PLV(t) \triangleq \left| {E\left[ {e^{j\Delta \phi (t)} } \right]} \right| $$

PLV is between [0,1]. The closer PLV is to 0, the more out of sync the phase of two signals will be; the closer PLV is to 1, the more synchronous the phase of two signals will be. Therefore, PLV can reflect the degree of synchronization between two signals by measuring their relative phases.

When calculating synchronization of cortical neural activity, a single source commonly mixed with the two signals may produce non-zero PLV (Aviyente et al. 2010). In this direct linear mixing, such two signals without phase delay may result in a large PLV. In order to eliminate the effect of linear mixing on phase locking, the phase lag index (PLI) is proposed, which quantifies the symmetry of relative phase around 0:

32 $$ PLI \triangleq \left| {E\left[ {sign(\Delta \phi (t))} \right]} \right| $$

PLI is between [0,1]. Only when the relative phase is 0 or π, PLI is 0. The nonparametric estimates of PLV and PLI can be approximated by averaging trials.

33 $$ P\hat{L}V_{trial} \triangleq \left| {\frac{1}{N}\sum\limits_{n = 1}^{N} {e^{j\Delta \phi (t)} } } \right| $$

34 $$ P\hat{L}I_{trial} \triangleq \left| {\frac{1}{N}\sum\limits_{n = 1}^{N} {sign(\Delta \phi (t))} } \right| $$

Non-parametric estimates of PLV and PLI were used to conduct phase locking analysis on the contained energy signals between the TPN and the two TNNs in three different phases of working memory, respectively.

## Results

### AMPA-caused oscillation was concerned with working memory

**Fig. 4:**
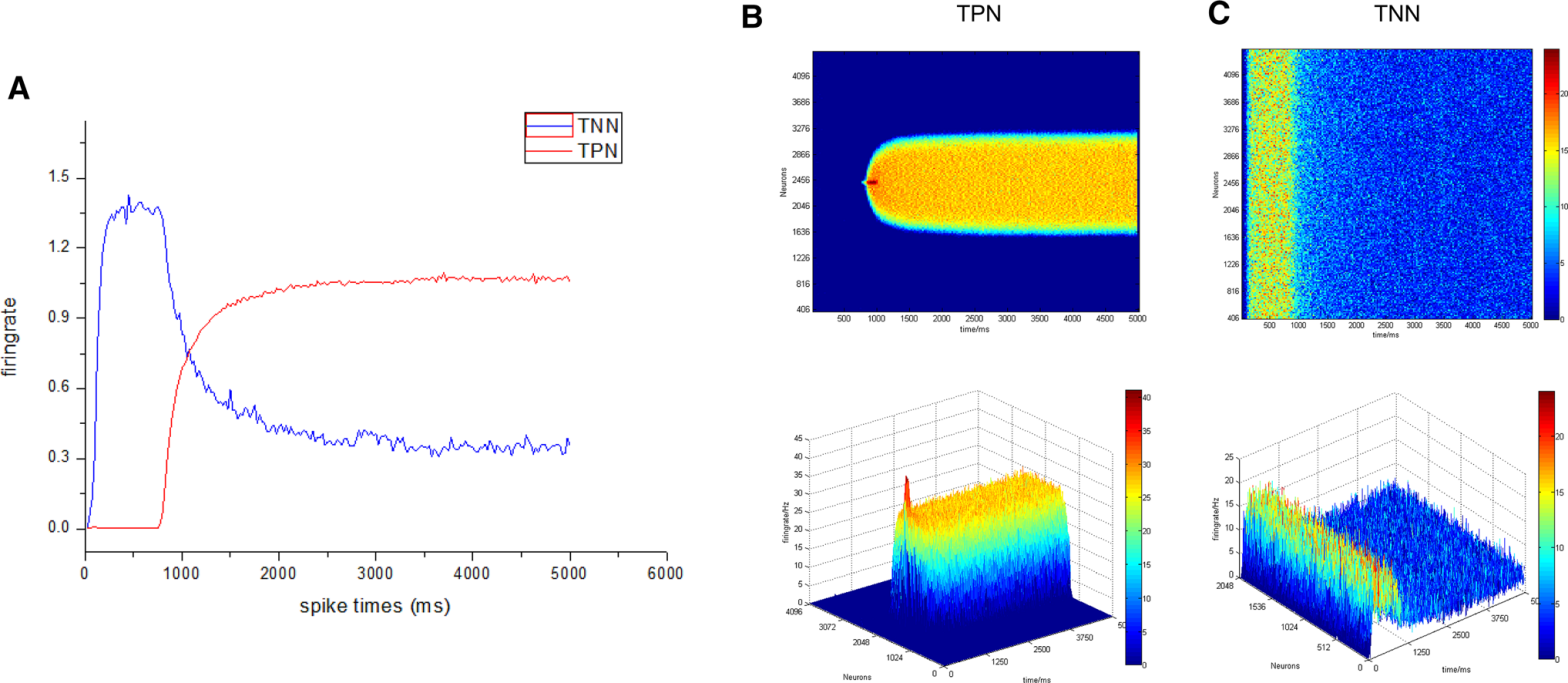
Basic anticorrelation behavior and firing results in the TPN–TNN network when AMPA conductance was set a small value. Gaussian weight parameter: $\sigma = 11.25,J^{ + } = 3.62,k5 = 90$, preferred direction: 180°. **a** Anticorrelation of firing rates in the TPN–TNN networks. The red curve indicates firing rates in the task-positive network (TPN), and the blue curve indicates firing rates in the task-negative network (TNN). **b **The scatter plot of firing rates in the TPN with density color temperature and 3D demonstration. **c** The scatter plot of firing rates in the TNN with density color temperature and 3D demonstration. The color temperature chart and the Z-axis are firing rates calculated by a 45 ms time window

Previous studies have shown that the TPN–TNN model based on synaptic mechanism could simulate the anticorrelation between the DMN and WMN (Cheng et al. 2020). Reproduced results of these studies (Fig. 4) showed the negative correlation between activities of the TPN and TNN in the TPN–TNN model. This is an important condition for exploring whether the positive and negative correlationship between the WMN and DMN can be integrated or transformed.

The basic results (Fig. 4) showed that the TPN–TNN network presented a unique mutual suppression phenomenon. Before 750 ms, the TPN did not receive the external current (I_ext in Eq. 3 was 0). This situation induced that synaptic currents of pyramidal cells in the TPN into both excitatory and inhibitory populations in the TNN were smaller than synaptic currents from the TNN into excitatory and inhibitory populations in the TPN. Then, inhibition of interneurons to pyramidal cells in the TNN was weaker than inhibition of interneurons to pyramidal cells in the TPN, while the excitatory neurotransmitter conductance in the TNN was larger than that in the TPN (see the supplementary material). With weak initial parameters, the TPN–TNN network presented such a special phenomenon that the TNN had a high firing rate in initial period without the external stimulus. This firing result could well accord with the activity mode of the DMN in resting state (Buckner et al. 2008; Raichle 2015).

**Fig. 5:**
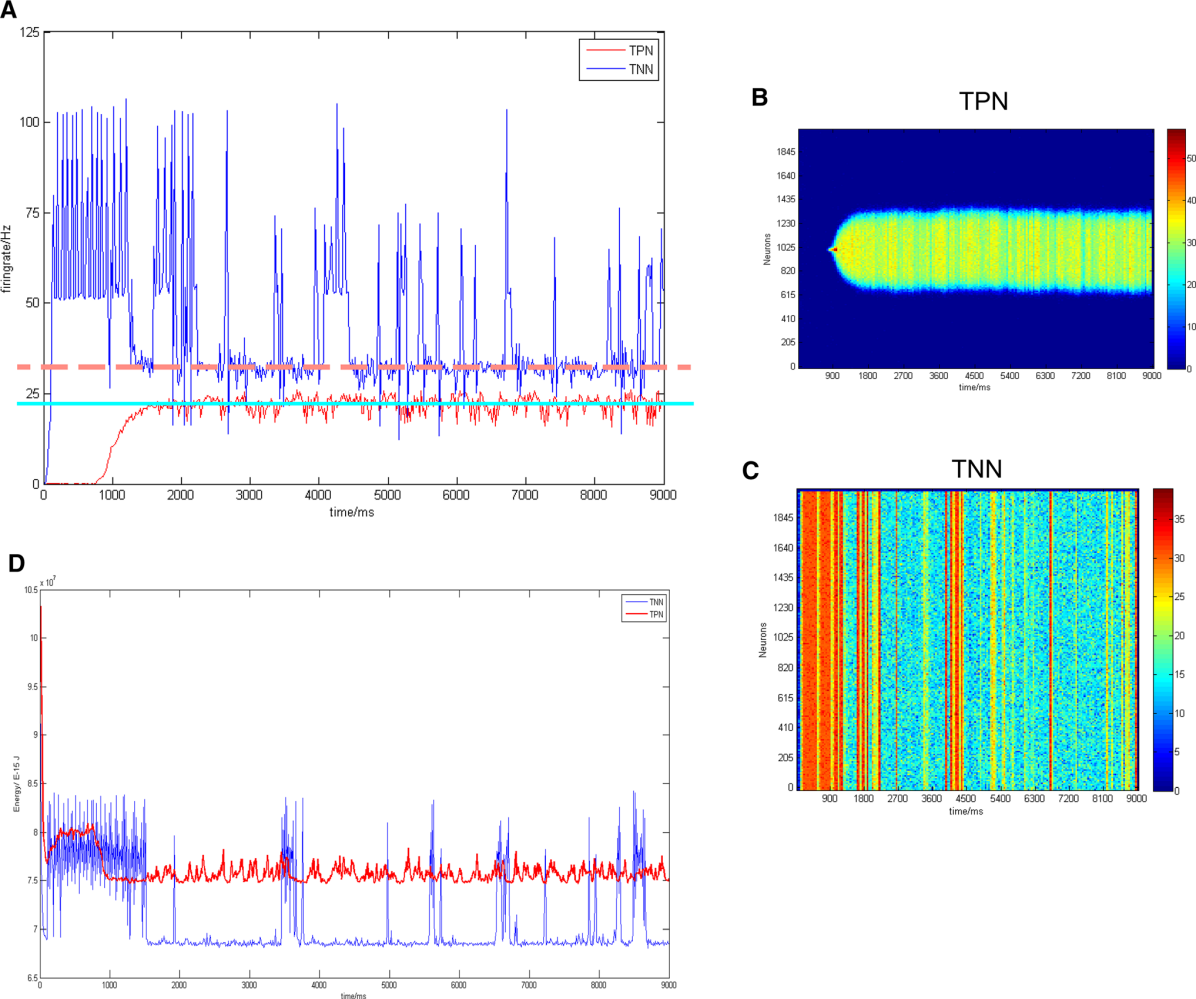
Firing rates in the TPN–TNN network when AMPA conductance had similar magnitude to NMDA conductance. **a** Firing rate curves of excitatory neurons in the TPN and TNN. Gaussian weight parameters: $\sigma = 11.25,J^{ + } = 3.62,k5 = 90$, preferred direction: 180°. Red dotted line is the baseline of firing rates in the TNN after stimulus withdrawal. The baseline value is 32.21 Hz. Bright blue solid line is the baseline of firing rates in the TPN after stimulus withdrawal. The baseline value is 21.32 Hz. **b** Scatter plot of firing rates in the TPN with density color temperature. **c** Scatter plot of firing rates in the TNN with density color temperature. **d** Contained energy in TPN and TNN. Red curve is the contained energy in the TPN, and the blue one is the contained energy in the TNN

However, in order to highlight the antagonistic effect, the strength of AMPA channels in the TPN (or in the TNN or between the TPN and TNN) was set a small value to eliminate synchronous oscillations in the TPN–TNN network. But setting small strength of AMPA channels hindered to further reveal the coupling mechanism between the DMN and WMN in a complete working memory process. Therefore, we adjusted the AMPA conductance in the network to the same order of the NMDA conductance, so as to introduce normal synchronous oscillations from AMPA. We set a higher value for AMPA conductance and keep other parameters and preferred direction as same as those in the basic trial (Fig. 4) to simulate neural activity in the TPN–TNN network (Fig. 5). We found acute oscillations in the firing rate (Fig. 5a), in scatter records (Fig. 5b, c) or in the contained energy (Fig. 5d). And the firing difference between neurons became significant. When calculating the firing rate, the Z-axis of 3D plot became no longer effective due to the prominent differences between neurons, so only the two-dimensional scattered plot with density color temperature was selected to redisplay the firing patten (Fig. 5b, c).

Relatively stable firing rates after 2000 ms were selected and averaged to obtain baseline values in the TPN and TNN, respectively. As could be seen from Fig. 5a, the TPN was still relatively stable after its firing rate approached the baseline value in the presence of AMPA, but the TNN presented a noisier firing rate curve. This phenomenon might be induced by the larger value of NMDA excitatory neurotransmitter conductance in the TNN, which led to a larger AMPA conductance proportionately. Hence, obvious oscillations appeared. Although the firing rate curve in Fig. 5a was reasonable, it was inappropriate to show the antagonism between the TPN and TNN.

The difference between Figs. 5a and 4a shows that the TPN–TNN network with larger AMPA excitatory synaptic connections had a more drastic oscillation. Although the firing rate curve (Fig. 5a) still showed the TPN–TNN anticorrelation, the relative positions of those two baselines altered. In Fig. 4a, the TNN baseline was lower than the TPN baseline; while in Fig. 5a, the TNN baseline was higher than the TPN baseline. This meant that, in the case of the same weight and inter-network conductance parameters, the mutual inhibition between the TPN and TNN with larger AMPA synaptic connections was weakened. AMPA had a significant impact on neuronal synchronous oscillation (Fuchs et al. 2001), making the default network unable to be well inhibited in task state.

It has been explored that the AMPA/NMDA conductance ratio could induce changes of neuronal dynamical states and oscillation patterns (Wolf et al. 2005). This method might be utilized to fine-tune some conductance ratio of AMPA/NMDA in order to adjust their baseline positions, making the TPN–TNN simulation closer to the coupling of the DMN and WMN (Fox et al. 2005; Cheng et al. 2020). To better present the basic anticorrelated phenomenon in the TPN–TNN model and appropriately retain the oscillation from AMPA introduction, the contained energy was calculated (Fig. 5d).

By adjusting the AMPA/NMDA conductance ratio in the TNN from 3.5/6.5 (Compte 2000) to 2.0/6.5, the contained energy curves in the TPN and TNN are shown in Fig. 5d. Since the contained energy was based on membrane potential, the adjustment of AMPA/NMDA ratio in the TNN could significantly modulate the oscillation in the contained energy and baseline positions in the TPN and TNN. Compared with Fig. 5a, after the adjustment of AMPA/NMDA ratio in the TNN, the contained energy baseline positions in the TPN and TNN were reversed. The negative correlation between the TPN and TNN became obvious after adjusting the TNN AMPA/NMDA ratio. Therefore, in the following sections, the AMPA/NMDA ratio within the TNN was set at 2.0/6.5 to ensure that the TNN could be well inhibited by the TPN during task periods.

The result also raises several questions. When population neurons had highly synchronized oscillations, what kind of index was more adequate to characterize anticorrelation between the TPN and TNN? We have observed negatively correlated activities in the TPN–TNN network. Then how to simulate positive correlation between the WMN and DMN observed during encoding and retrieval stages in the working memory task? Is it possible to simulate both positive and negative correlations in the same theoretical model by changing neurotransmitter parameters and internal weights among neurons? If so, how could the two types of correlations be switched in the same TPN–TNN model? These questions would be answered in following paragraphs.

### Modification of NMDA channels between the TPN and TNN regulated the transmission of neuronal information.

Different correlations between the DMN and WMN have been observed in maintenance and retrieval phases of a working memory task with fMRI (Piccoli et al. 2015). For theoretical research, we want to know what kinds of factors which enable the networks to switch from the maintenance state to the retrieval state. In another words, our question is how to change the activity pattern in the TPN–TNN model when it behaved at the stable baseline stage (Fig. 5). We attempted to use two methods. One was to repeat the same external stimulus and the other was to modify the NMDA conductance between the TPN and TNN, in order to find a reasonable explanation for changes of functional connections and correlations between brain regions associated with the phase switch of working memory.

#### The effect of repeated stimulus

**Fig. 6:**
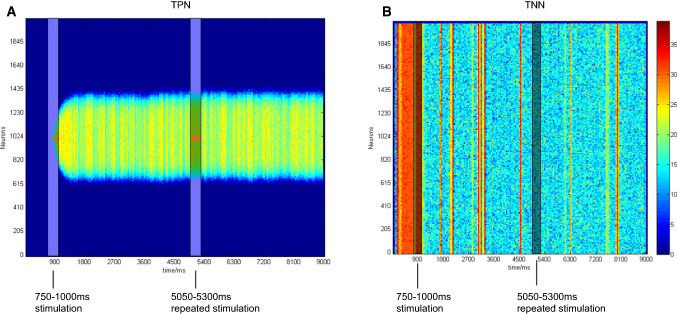
Firing rates of excitatory neurons in the TPN and TNN when a repeated preferred direction stimulus was presented. **a** Scatter plot of firing rates in the TPN with density color temperature. **b** Scatter plot of firing rates in the TNN with density color temperature. Preference direction: 180°. Gaussian weight parameter: $\sigma = 13.25,J^{ + } = 3.62,k5 = 95$. The first stimulus was presented during 750–1000 ms. The same stimulus was repeated during 5050–5300 ms. Stimulation periods had been respectively marked with reverse color boxes

During simulation, a stimulus with a preferred direction of 180° was inputted into the TPN from 750 to 1000 ms as an indicator of the memorized position. During the memory maintaining phase, the same stimulus was re-presented from 5050 to 5300 ms. Figure 6 showed firing rates of neurons in the TPN and TNN, respectively. In this case, we did not modify any neurotransmitter parameters at any time point in the whole simulation process. It could be observed that the repeated stimulus did not affect the activity pattern of the TPN–TNN network as long as the network had reached a stable and strong population firing state. The only difference was that during the repeated stimulus period (Fig. 6a, 5050–5300 ms), the firing rate in the TPN increased, while the stable inhibitory state in the TNN would not be re-activated by the repeated stimulus. The results suggest that the repeated stimulus did not change the neural activity patterns in the TPN and TNN simultaneously.

#### The effect of modifying NMDA conductance between the TPN and TNN

**Fig. 7:**
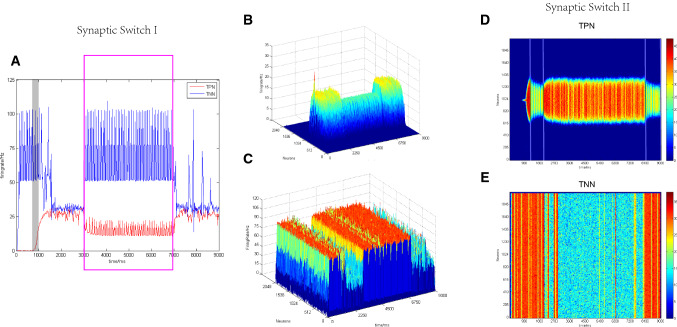
**a**–**c** Firing rates in the TPN and TNN for switch I and switch II, respectively. **a** Firing rate curves in the TPN and TNN under the condition of switch I. The red curve is the firing rate of TPN. The blue curve is the firing rate of TNN. The gray box means the stimulation period (750–1000 ms), and the magenta box indicates the period in which NMDA channels between the TPN and TNN were switch-off (3000–7000 ms). Preference direction: 180°, Gaussian weight parameter: $\sigma = 13.25,J^{ + } = 3.62,k5 = 95$. **b**, **c** 3D scatter plot of firing rates in the TPN and TNN under switch I, respectively. **d, e** 2D scatter plot of firing rates in the TPN and TNN with density color temperature under switch II. Periods marked by straight lines in (**d)** were the inter-network NMDA switch-off intervals (1000–2000 ms, 8000–9000 ms). Those intervals were the same in (**e**)

We studied two methods to modify excitatory synaptic conductance between the TPN and TNN, in order to find out a possible switching mechanism from the maintenance phase to the retrieval phase in the working memory process. One method was to switch off NMDA channels between the TPN and TNN (setting NMDA conductance as zero) during a maintaining period (from 3000 to 7000 ms), then NMDA channels returned to normal states after this period. We observed changes of network activity patterns during the whole maintaining period. The other method was to switch off NMDA channels between the TPN and TNN during 1000–2000 ms and the period after 8000 ms, respectively. NMDA channels were kept normal values during 2000–8000 ms in which the TPN–TNN model exhibited anti-correlated activities. Since AMPA only affected the oscillation of population neurons, AMPA channels were not regulated in this study. These two switch methods of NMDA channels between the TPN and TNN were hereafter called as switch I (Fig. 7a–c) and switch II (Fig. 7d–e).

Figure 7 showed firing rate curves and scatter plots under the two NMDA switches. Activity patterns in both the TPN and TNN changed at the starting point of the NMDA switch I (3000 ms, Fig. 7a–c) due to the truncation of NMDA channels between them. For the lack of the inter-network NMDA projections from pyramidal cells to interneurons, the mutual inhibition of TPN and TNN was significantly weakened. In this case, the activities in the TPN and TNN were more dependence on their intrinsic neurodynamics. The firing in the TNN became stronger during switch-off period (3000–7000 ms) because of its relatively higher internal synaptic parameters. After 7000 ms, the inter-network NMDA projections recovered to normal values, i.e. NMDA switched on again. The whole network turned to the activity situation observed before 3000 ms. However, in the case of NMDA switch II, only the neural activity in the TPN changed accordingly due to the switch (Fig. 7d–e), while the firing activity in the TNN did not decreased after the stimulation period (750–1000 ms) until NMDA connections between the TPN and TNN switched on. This indicated that NMDA switch II could not change the activity patterns in the TPN and TNN simultaneously.

During switch I, the simultaneously changed activities in the TPN (representing the WMN) and TNN (representing the DMN) were closer to the conversion of activity patterns between different phases of working memory. Therefore, we selected the simulated data of NMDA switch I to calculate the contained energy for further analysis.

**Fig. 8:**
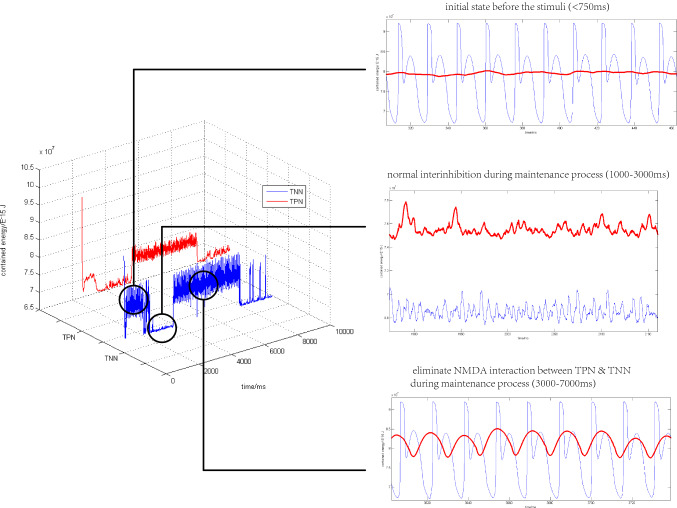
The contained energy curves in the TPN and TNN under NMDA switch I. The whole network received a external stimulus during 750–1000 ms. NMDA channels between TPN and TNN were switched off during 3000–7000 ms (right below) and switched on in the rest of simulation time. Preference direction: 180°, Gaussian weight parameter:$\sigma = 13.25,J^{ + } = 3.62,k5 = 95$

As shown in Fig. 8, after switching off the NMDA synaptic conduction between the TPN and TNN (3000–7000 ms), the contained energy in each of the two networks became more rhythmic (PLV = 0.9993, PLI = 0.1740, the right bottom subplot of Fig. 8) than that during other periods. By switch I, when the TNN negatively correlated with the TPN (1000–3000 ms), the whole network consumed more energy (i.e. contained energy decreased) because of their mutual inhibition, corresponding to a higher TPN firing rate curve (Fig. 7a) and a lower TPN contained energy curve in (Fig. 8). However, after switching off NMDA channels between the TPN and TNN (3000–7000 ms), the contained energy in the TNN returned to the oscillation situation when no external stimulus was applied and the contained energy in the TPN appeared new oscillations that had not been observed previously. The simultaneous appearance of oscillations in the TPN and TNN might have relevance with the positive correlation phenomenon between the WMN and DMN reported in the fMRI experiment (Piccoli et al. 2015).

#### The effect of NMDA conductance anomalies

It had been confirmed in Sects. 3.2.1 and 3.2.2 that the same repeated stimulus could not significantly enhance TPN activities in the model with the coupled TNN, while that the switch on and off of NMDA channels between two networks had an important impact on activities in the TPN–TNN network. During the switch I method, the strength of NMDA conductance was set as zero (switch off condition) or was kept as a normal value (switch on condition). It was still not sure whether the activity in the TPN and TNN would concurrently be changed with adjustment of NMDA conductance values, especially strengthening NMDA gating connection, instead of the 0–1 type NMDA switch. Therefore, in this section, we investigated the effect of significantly higher NMDA conductance on activity patterns in the TPN and TNN and their responses to the external stimulus.

**Fig. 9:**
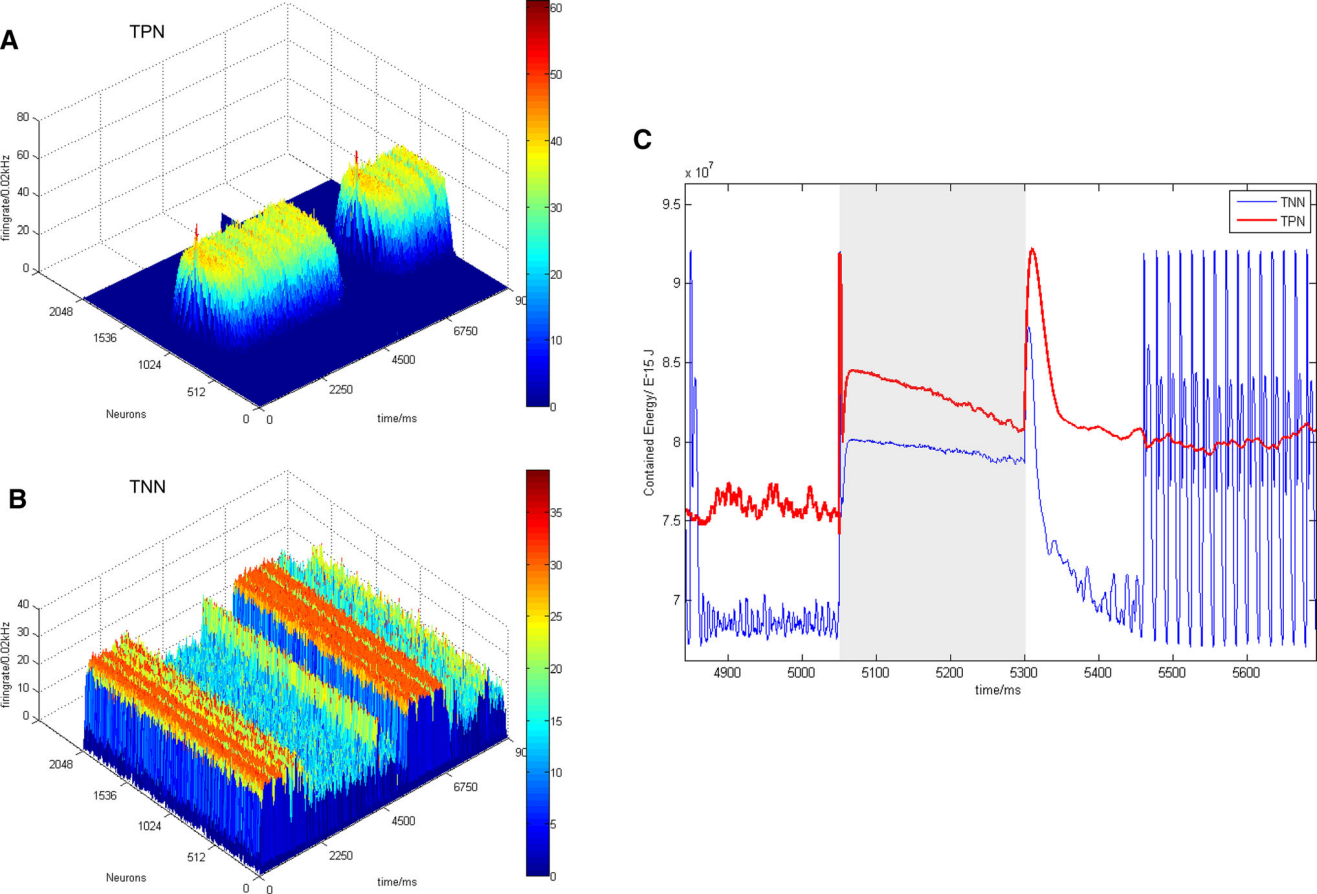
Results of interaction between the TPN and TNN with abnormally high values of NMDA conductance and reapplied stimulation afterwards. **a**, **b** The 3D scatter plot of firing rates in the TPN and TNN. **c** The contained energy curves in the TPN and TNN during 4900–5600 ms with a grey area showing the time period (5050–5300 ms) NMDA conductance value was extremely high. Preferred direction: 180°. Gaussian weighting parameters: $\sigma = 13.25,J^{ + } = 3.62,k5 = 95$

The NMDA conductance between the TPN and TNN was set ten times as great as the normal value during 5050–5300 ms, but was kept the normal value in other periods. In addition to the initial stimulus in 750–1000 ms, a second identical stimulus was presented in 6550–6800 ms (Fig. 9).

It was found that both TPN and TNN activity patterns were changed after a large increase in NMDA conductance (Fig. 9a). Particularly, the TPN directly stopped firing with the abnormally large NMDA conductance (5050–5300 ms). After that, with NMDA conductance returning to the normal value, the TPN did not fire until the same stimulus was re-represented during 6550–6800 ms. This repeated stimulus evoked a similar activity pattern in the TPN to the response of the first stimulus (750–1000 ms). The large NMDA conductance halted the normal firing activity in the TPN (Fig. 9a), which allowed the otherwise inhibited TNN to become active again (Fig. 9b) and returned the entire network into a resting-like pattern. The possible reason was that the excitatory synapses were so strong that the inhibition given by the inhibitory synapses could not be comparable, resulting in synaptic disconnections between neurons throughout the network.

As could be seen from the contained energy curves in Fig. 9c, the abnormally high NMDA conductance made contained energy in both the TPN and TNN increase, while the both contained energy curves became gradual and mild when firing activity in the TPN stopped and firing activity in the TNN enhanced. This phenomenon reflected that an excessively high NMDA conductance could interrupt energy consumption (Kristiansen and Meador-Woodruff 2005) so as to interrupt an entire working memory process. After 5300 ms, the strength of NMDA conductance returned to the normal value as same as that before 5050 ms. Both the TPN and TNN showed a peak-like fluctuation in contained energy. After consuming more, the contained energy of the TNN entered into an oscillation form, while the contained energy of the TPN fluctuated slightly around a stable value. Comparing the upper right subplots of Figs. 8 and 9c after 5300 ms, it could be observed that the whole TPN–TNN network returned to the initial firing state without stimulation. Combining the three graphs in Fig. 9, it represented that a new working memory process flagged by a repeated stimulation (after 6550 ms) after a period of excitatory neurotransmitter anomalies was equivalent to restarting the memory, without any phenomenon of memory recall and reinforcement.

### Working memory processes with sequential stimuli with different preferred directions

The aforementioned modulation of NMDA represented the robustness of this mutual inhibitory network structure by the direct repetition of external stimuli (“The effect of repeated stimulus” section) and the autonomous repetition of internal switch-on and -off neurotransmitter gating channel between the DMN and WMN (“The effect of modifying NMDA conductance between the TPN and TNN” and “The The effect of NMDA conductance anomalies” sections). In order to deeply understand functional roles of the DMN in working memory, we inputted two external stimuli with different preferred directions to the TPN at two different moments sequentially, to discover how the two sequential stimuli affected working memory process, and to verify the necessity of the DMN in concert with the WMN.

**Fig. 10:**
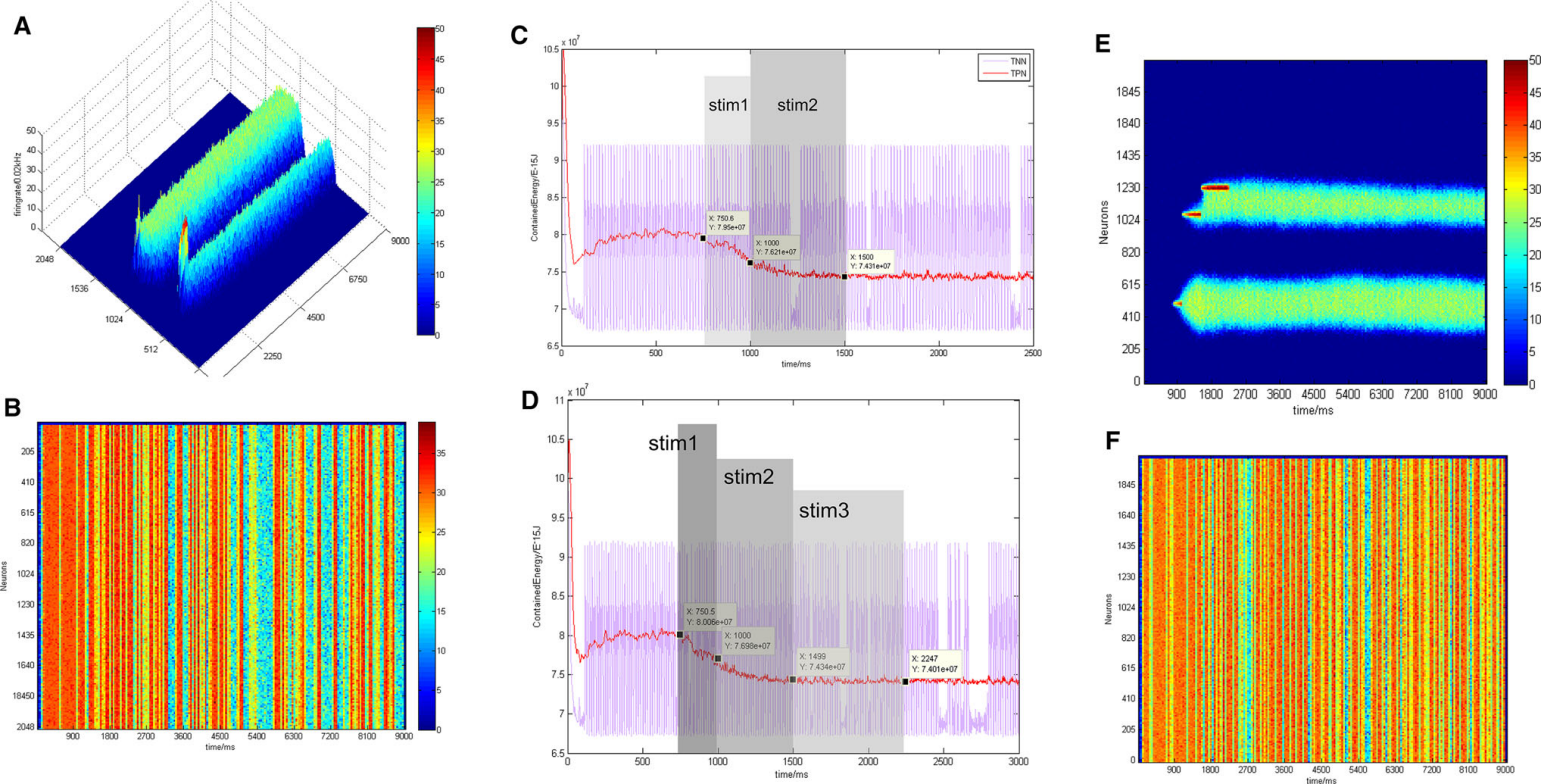
Simulated results of sequential stimuli. **a**, **b**: scatter plots of firing rates in the TPN and TNN with two sequential stimuli. **c **The curves of contained energy in the TPN and TNN when the two sequential stimuli were presented. Preferred directions and application time periods of the two sequential stimuli were: Stim1: 200° 750–1000 ms; Stim2: 100°1000–1500 ms. Gaussian weighting parameter: $\sigma = 9.25,J^{ + } = 3.62,k5 = 95$. D: The curves of contained energy in the TPN and TNN when the three stimuli were presented sequentially. **e**, **f** Scatter plots of firing rates in the TPN and TNN when the three sequential stimuli were presented. Preferred direction and application time periods of three sequential stimuli were: Stim1: 90° 750–1000 ms; Stim2: 190° 1000–1500 ms; Stim3: 220° 1500–2250 ms. Gaussian weighting parameter:$\sigma = 9.25,J^{ + } = 3.62,k5 = 90$

When the two stimuli were inputted into the TPN sequentially (Fig. 10a, b), the number of activated TPN pyramidal neurons during the stim2 period (1000–1500 ms) was significantly higher than that during the stim1 period (750–1000 ms). The firing rate of TPN neurons in the stim2 period was higher than that in the stim1 period. However, during the delay period, the firing rate of population neurons activated by stim2 was not higher than that of stim1 (Fig. 10a). Comparing with the results of one single stimulus (Fig. 5c), the TNN was not less inhibited by the two sequential stimuli. To ensure the firing pattern induced by stim2 was kept during the maintenance-like phase, the Gaussian weight parameter σ between excitatory neurons in the TPN was set a smaller value compared to that in previous simulations. Thus, because fewer neurons in the TPN were activated in the two-stimulus trials than in one-stimulus trial, inhibition of the TNN was reduced when stim1 was inputted. Oscillations in the TNN were more intense than those with one single stimulus, but inhibition in the TNN still occurred (Fig. 10b).

As could be seen in Fig. 10c, the TPN consumed almost all of its stored energy in the presentation of stim1 period from 750 to 1000 ms ($\Delta E \approx 3.3\;{\text{nJ}}$); while in presentation of stim2 period from 1000 to 1500 ms, there was less energy consumption ($\Delta E \approx 1.9\;{\text{nJ}}$). During the two stimuli presentation period, the TNN kept stable energy values and no significant changes in firing activity (background-like violet oscillation curves in Fig. 10c). This meant that the pattern of energy consumption and neuronal firing activity throughout a working memory simulation trial was determined during the 750–1000 ms stim1 period.

With almost identical parameters shown above, the model was difficult to "remember" the third stimulus when three stimuli were inputted into the network sequentially. The sustained firing population induced by stim3 probably was fused to the sustained firing population caused by stim2 (Fig. 10e), while the TNN did not get enough inhibition. Even when the direction of stim3 was far away from the direction of stim2, we had not succeeded to keep a sustained firing pattern evoked by stim3 during the delay period. Moreover, it was seen from the contained energy curves (Fig. 10d) that the energy consumption of the TPN was about 3.08 nJ, 2.64 nJ, and 0.33 nJ for stim1, stim2 and stim3, respectively. Arguably, for this set of direction selective weighting parameters ($\sigma = 9.25,\;J^{ + } = 3.62$), the TPN–TNN model could only remember at most two sequential stimuli in one trial. Simulation results of three sequential stimuli (Fig. 10d–f) indicated that only within a certain range of energy consumption could allow the network to add new stimuli as memory components. It was difficult for a TPN–TNN network with determined energy consumption to change its neural activity. In addition, during a working memory process with involvement of the DMN, a successful addition of subsequent stimuli would shift the center of the neuronal community activated by previous stimulus (Fig. 10e), but would not stop the original working memory process. The coupling of the DMN could make working memory more stable but also more immutable or robust.

### Simulation of three phases in a working memory process

The synaptic model originally proposed by Wang and Compte (2000; Wei et al. 2012) has well demonstrated maintaining-like activity in working memory. It has also been shown that the TNN as a default network coupled with the TPN was a good way to achieve the anticorrelation between the WMN and the posterior cingulate brain region (a part of the DMN) during the working memory maintenance phase (Cheng et al. 2020). However, these models neither took into account encoding and retrieval phases of working memory, nor considered positive correlation between the WMN and DMN during these two phases. Therefore, we began to consider whether a model could be implemented for an entire working memory process that completely contains encoding, maintenance, and retrieval phases.

The topic of this article is to explore whether positive and negative correlations between the DMN and WMN at different phases would be integrated at the energy level. BOLD signals in the fMRI working memory study showed diametrically opposed correlations between the DMN and WMN in maintenance phase and in encoding or in retrieval phase of working memory (Piccoli et al. 2015; Vatansever et al. 2017). Since the negative and positive correlations between the DMN and WMN are manifested at different stages of the same cognitive task and also in functional connectivity between different brain regions, it was possible that these two correlations could be switched each other to realize a continuous working memory process without break intervals. In order to theoretically explore this possible switching and combination of negative and positive correlations between the WMN and DMN, we simulated a working memory process that completely contained three phases.

The specific network architecture was shown in Fig. 2 and “Methods” section, which consisted of two TNNs (TNN1 and TNN2) and one TPN. In order to realize the positive correlation that might occur during encoding and retrieval phases, excitatory neurons in the TNN2 had the same directional weights as those for excitatory neurons in the TPN. In a simulated working memory trial, an external stimulus with a preferred direction was inputted into the TPN during 750–1000 ms. After that there was a maintenance period and a retrieval period (from 800 to 9000 ms).

The contained energy in the TPN and TNN had better embodied characteristic oscillations than did firing rates in the NMDA switch I simulation trial (Fig. 8). In that case, we applied NMDA switch I to simulate inherent changes of the network when the working memory phase was shifted. Once the working memory phase changed, the connections between TPN and TNNs would simultaneously alter. Contained energy was the most important index to illustrate the oscillation differences of three networks in three phases.

In some simulation trials, simultaneous switching of both AMPA and NMDA channels between the TPN and TNN was also attempted. It was found that AMPA only controlled the oscillatory strength of the overall network activity, and its effect on other aspects of neuronal activity was not significant. Therefore, in this section, we preserved normal AMPA channels and only used NMDA switch I (Figs. 7, 8) to respectively switch on or off the connections of the TPN (representing DLPFC) with the TNN1 (representing PCC) or with the TNN2 (representing IPL) in three different phases of a complete working memory process.

Parameters of the model and time intervals of three phases are shown below. Detailed internal parameters of the model are shown in the supplementary parameter table. Gaussian weight parameters: $\sigma = 11.25,\;J^{ + } = 3.62,\;k5 = 95$. Preferred direction of the external stimulus: 180°. The external stimulus was applied in 750–1000 ms. Encoding phase: 750–1750 ms. In this encoding period, NMDA conductance was kept a normal value between the TPN and TNN2, while NMDA conductance was set zero between the TPN and TNN1. Maintenance phase: 1750–8000 ms. In this maintenance period, NMDA conductance was kept a normal value between the TPN and TNN1, while NMDA channels between the TPN and TNN2 were switched off (the strength of NMDA conductance as set as zero). Retrieval phase: 8000–9000 ms. In this retrieval period, NMDA channels were switched on at a normal level between the TPN and TNN2, while they were switched off between the TPN and TNN1. All AMPA and GABA currents in the TPN–TNNs model did not vary during all three phases.

**Fig. 11:**
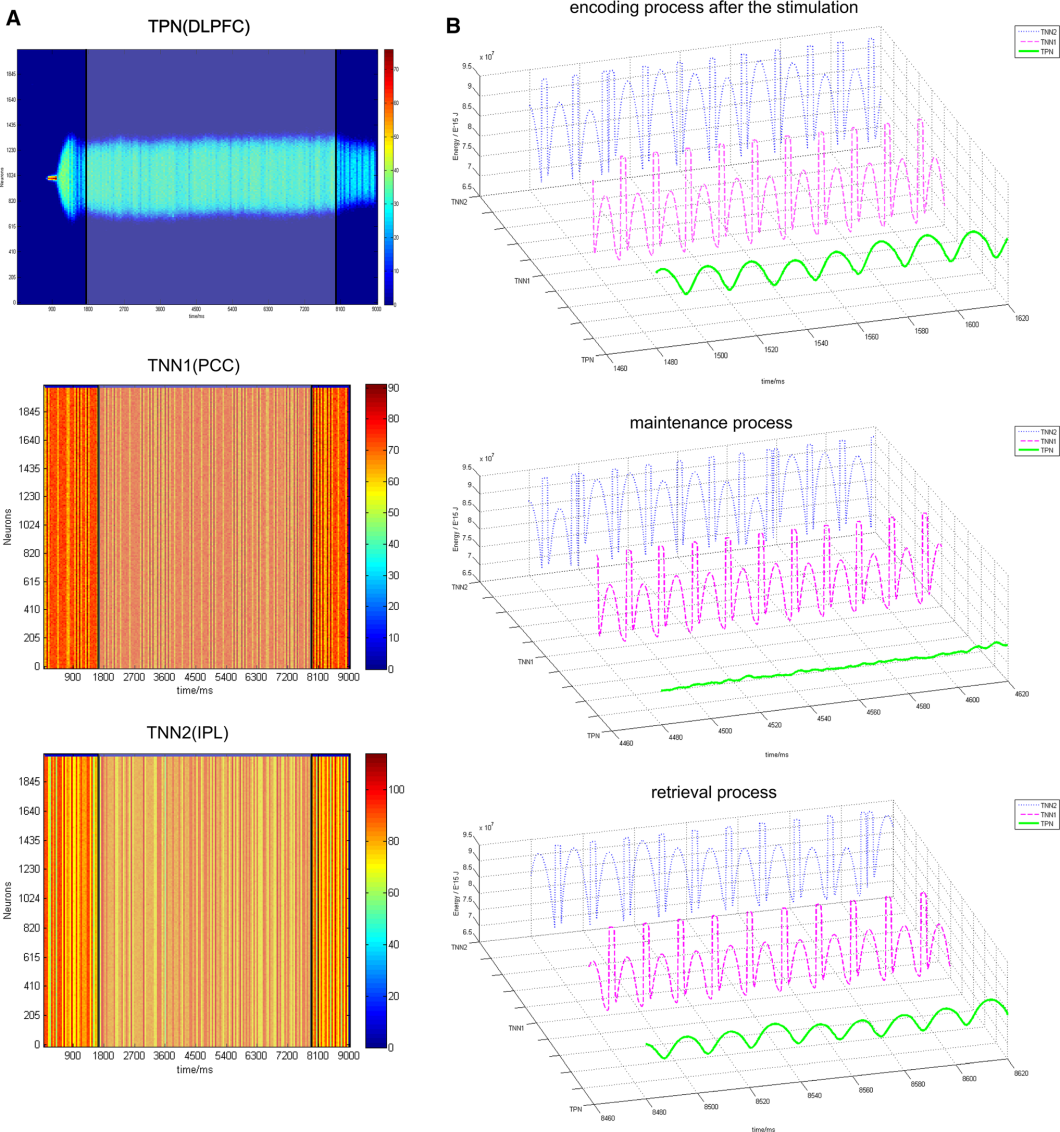
Firing activity and contained energy in a complete working memory process. **a** Scatter plots of firing rates in TPN and two TNNs with density color temperature. The semi-transparent white box marks the maintenance phase. Black lines on both sides of the white box are the time points of encoding → maintenance (1750 ms), maintenance → retrieval (8000 ms). **b** Contained energy curves of the three networks by intercepting time intervals of encoding, maintenance, and retrieval phases. The blue dashed curves indicate contained energy in the TNN2; the magenta dashed curves represent contained energy in the TNN1, and the green solid curves are contained energy in the TPN

As could be seen in Fig. 11a, the TPN could not inhibit the two TNNs well. However, this result indicated that the BOLD signal correlation between the WMN and DMN observed in fMRI experiments was not completely characterized by the high or low baseline value of firing rates. Due to the consistency between the BOLD signal and contained energy (see “Methods”), the contained energy would reflect more characteristics of neural activity than does the firing rate. The oscillations of contained energy were significantly different in the TPN, TNN1 and TNN2 (Fig. 11b). Oscillations in the TPN disappeared during the maintenance phase from 1750 to 8000 ms (Fig. 11b, middle panel). This disappeared oscillation was probably related to NMDA connections between the TPN and TNN1, NMDA disconnections between the TPN and TNN2 during this maintenance period. In order to quantitatively measure oscillation patterns in the three networks, phase locking analysis was carried out based on the contained energy signals between every two networks.

The phase locking analysis is often used in fMRI studies and is a good method to measure phase synchronization of two BOLD signals recorded from two different brain areas. The two indexes of PLV (Phase Locking Value) (Aydore et al. 2013) and PLI (Phase Lag Index) (Stam et al. 2007) have been used here for analyzing phase synchronization of the contained energy between TPN and TNN1, TPN and TNN2, TNN1 and TNN2, separately in three phases of working memory. Since phase differences between three networks were small, PLV values between every two networks at each of three phases were very high (see Table 1). In that case, PLI was calculated at the same time. PLI mainly analyzes the phase symmetry of two signals, thus being able to amplify some tiny phase differences so that the discrimination of phase locking property could become obvious.

**Table 1:** PLV and PLI in different working memory phases between the three networks two by two. The contained energy of TPN in maintenance phase was a small noise-like fluctuation (Fig. 11b), for which the computed PLV and PLI were invalid

Working memory process	Networks	PLV (Phase locking value)	PLI (Phase lag index)
Encode (after stim)	TPN&TNN1	0.9991	0.7384
	TPN&TNN2	0.9991	0.7306
	TNN1&TNN2	0.9969	0.5178
Maintenance	TPN&TNN1	–	–
	TPN&TNN2	–	–
	TNN1&TNN2	0.9968	0.5313
Retrieval	TPN&TNN1	0.9996	0.8485
	TPN&TNN2	0.9996	0.8181
	TNN1&TNN2	0.996	0.4871

**Fig.12:**
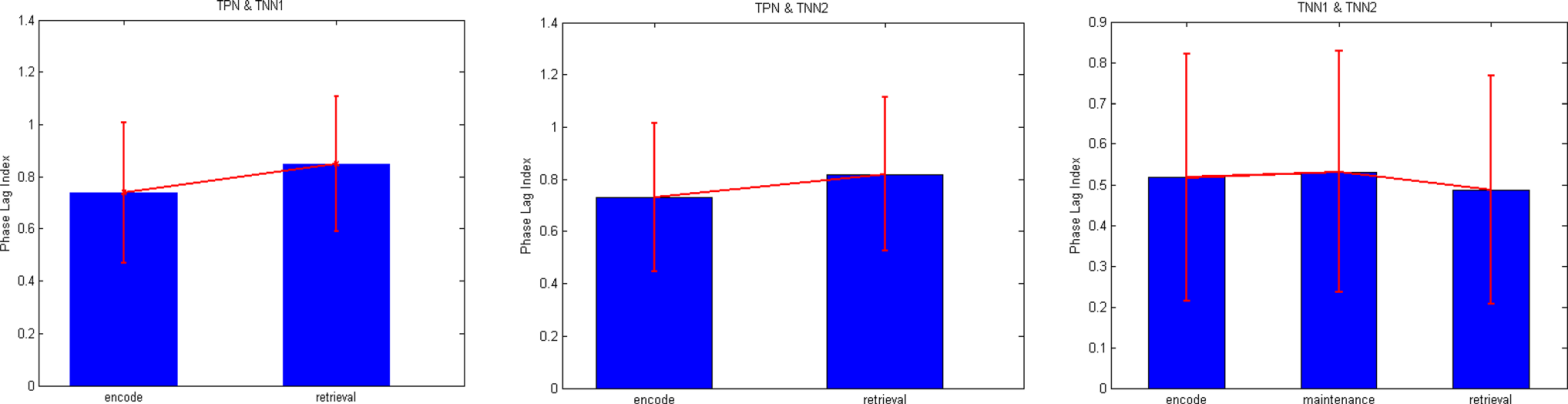
PLI at different working memory phases between three networks. Invalidation results of TPN and TNN1 and TPN and TNN2 was directly omitted here

From Table 1 and Fig. 12, it could be found that both PLI of TPN and TNN1 and PLI of TPN and TNN2 were somewhat higher during the retrieval phase than during the encoding phase. This result was consistent with the phenomenon of working memory enhancement in retrieval phase (Hsieh and Ranganath 2014; Karlsgodt et al. 2005). Phase locking between TNN1 and TNN2 was common in all three phases, but there was a small decrease in PLI during the retrieval phase comparing with PLI in the encode or maintenance period. The PLI between TPN and TNN1 and the PLI between TPN and TNN2 were high during encoding and retrieval phases. However, the contained energy in the TPN showed a flat signal without oscillations during the maintenance phase (Fig. 11b). So PLI during the maintenance phase was unable to be calculated as a valid value. In brief, the phase locking differences between maintenance and the other two phases might correspond to the distinction of negative correlation (Cheng et al. 2020; Andreou et al. 2018; Hsieh and Ranganath 2014; Dixon et al. 2016b) in maintenance and positive correlation in encoding and retrieval (Fox et al. 2005) between the WMN and DMN observed in their BOLD signals.

Theoretically, the simulated results using the two TNNs model could indeed be achieved by only using the model coupled with one single TNN, as demonstrated by the results of NMDA switch I (Figs. 7,8). The network model with one TNN created a kind of neuronal activity pattern shift that could be used to explain the transition of working memory from maintenance to retrieval phase. However, the TNN in the single TNN model was directly inhibited after the stimulus offset (Fig. 8), so there was no way to induce effective oscillatory properties during encoding phase, a short time after the stimulus offset. Furthermore, given that positive and negative correlations were observed in different brain regions (Piccoli et al. 2015; Dixon et al. 2016b), and that both PCC and IPL are subnetworks of DMN, it was more reasonable to use two mutually coupled TNNs as two subnetworks of the DMN.

The simulated results in three phases of working memory indicated that negative or positive correlation in experimental BOLD signals could correspond to contained energy of neuronal population with or without phase synchronization. The negative and positive correlations between the DMN and WMN showed integration and convertibility on energy representation.

#### Discussion

This article studied dynamical activity in a coupled TPN–TNN network to demonstrate functional roles of the DMN in a working memory task. First, we found that AMPA channels were able to cause significant synchronous activity in the networks (Fig. 5). The contained energy was able to represent significant oscillatory population activity (Fig. 5d). Second, we confirmed that changes in NMDA synaptic conductance between the TNN and TPN could switch neural activity among multiple patterns in the network (Figs. 8, 9, 10). In particular, the method of NMDA switch I, which sequentially disconnected and reconnected NMDA channels between the TPN and TNN after the stimulus offset (Fig. 8), resulted that the evoked firing activity was similar to the neuronal activity pattern shifting from maintenance phase to retrieval phase in the working memory task (Piccoli, et al. 2015). It was suggested that NMDA switch I might be an important neural mechanism to switch neural activity from maintenance phase to retrieval phase in working memory. In addition, sequential presentations of two stimuli with different preferred directions showed important roles of the TNN coupled with the TPN. The simulated results indicated that the number of sequentially mnemonic stimuli was related to the energy consumption determined by the network internal parameters (Fig. 10). Finally, we found that the three phases of working memory might correspond to functional connections between the WMN and different brain regions of the DMN with the collateral evidence of Piccoli.2015 (Piccoli et al. 2015). The maintenance phase was corresponding to NMDA channel switch-on in TPN–TNN1 (representing DLPFC-PCC) connections, while encoding and retrieval phases were corresponding to NMDA channel switch-on in TPN–TNN2 (representing DLPFC-IPL) connections. Neuronal oscillations of these functional connections showed differences in terms of phase synchronization (Figs. 11, 12). These findings demonstrated that the coupled activity of the DMN and WMN could not be described simply as either positive or negative correlation. These correlations were more likely to correspond with rhythmic activity characterized with different degree of synchronization in the DMN and WMN. From the view of the energy, the positive and negative relationships between the activity in the DMN and WMN could be integrated and converted each other.

The DMN is strongly linked to working memory (Vatansever et al. 2017). Abnormal activity in the DMN has been widely recognized as an important characterization of depressive states or cognitive abnormalities (Sheline et al. 2009; Broyd et al. 2009). It is also reported that working memory dysfunction with abnormal EEG signal fluctuations has frequently been observed in schizophrenia (Anticevic et al. 2013) and post-traumatic stress syndrome (PTSD) patients with typical cognitive deficits (Schweizer and Dalgleish 2011). Successive positive and negative correlations between the DMN and WMN were related to different functional connections between cortical regions during a complete working memory process. The inferior parietal lobe (IPL), positively correlated with the WMN, belongs to the medial temporal subsystem of the DMN; while the posterior cingulate cortex (PCC), negatively correlated with WMN, belongs to the dorsal medial subsystem of the DMN. These two subsystems serve different cognitive functions. The medial temporal subsystem, including hippocampus, is associated with autobiographical memory as well as event prediction, and the dorsal medial subsystem is associated with understanding of others and empathy (Andrews-Hanna et al. 1316). Resting-state functional connections between these two default network subsystems were found significantly attenuated in brains of major depression patients (Yan et al. 2019) and PTSD patients (Miller et al. 2017), triggering typical conflict avoidance symptom of these two psychiatric disorders. In addition, different brain regions can be identified by their different activity patterns observed in high precision fMRI even though they are very close (Scholz et al. 2009). This experimental evidence suggested that different regions in the same brain network could represent different activity patterns. Thus, from a physiological point of view, it is plausible that positive and negative correlations between the DMN and WMN appeared alternatively in a complete working memory process. It has also reflected that the coupled relationship between the DMN and WMN was more than just negative correlation (Dixon et al. 2016b).

By adjusting the AMPA term (Eqs. 10,12), which has been minimized in a previous paper (Cheng et al. 2020), to a value similar to the value of the NMDA term in the present study, neural activity in the TPN–TNN network displayed strong oscillatory properties (see Fig. 5). It has been shown that the AMPA channel plays an important role as an oscillatory factor in the synaptic communication between neurons (Kitanishi et al. 2015), as well as in the rapid high-frequency firing of epileptic signals (Bialer et al. 2007). It was reported that the membrane potential of a neuron with fast and high-frequency firings showed directional selectivity (Carver et al. 2008), consistent with the directional selectivity of the weights set in the WMN (Compte 2000). Therefore, it is feasible to characterize activities of high-frequency firing neurons using their membrane potentials. However, the model in this article was a coupled structure of two networks (Fig. 1a), or even of three networks (Fig. 2), with 2048 excitatory neurons and 512 inhibitory neurons in each network. At the level of a large neuron population, taking the averaged membrane potential over all neurons as local field potential (LFP) was not entirely appropriate and was difficult to interpret theoretically. Therefore, we used the membrane potential of each neuron to calculate the contained energy in each of the networks (Eqs. 27,28). Most of the contained energy for neural activity in brain (represented by ATP stored in mitochondria) is expended on synaptic activities (Harris et al. 2012). Moreover, the contained energy calculated in the TPN–TNN network was in agreement with the BOLD signal computed from firing rates of neurons (Fig. 3). Energy, a physiologically specific parameter, can provide a valid analysis for high-intensity oscillations caused by AMPA channels.

Since it is necessary to prove whether activities in the DMN and WMN with neural oscillations were unaltered in three working memory phases, it is important to confirm what kind of approach could be used to distinguish neural activities among three phases. The majority of experimental literature (Jaeggi et al. 2008; Hsieh et al. 2011; Heuer and Schubö 2016) used a cue stimulus presented at the end of maintenance phase to indicate subjects to shift working memory from the maintenance phase into the retrieval phase. However, from the theoretical view, it is not clear whether the presented cue stimulus just works as an external stimulus that could evoke activity changes in the TPN–TNN network to shift the working memory process from maintenance to retrieval phases. Alternatively, the presented cue stimulus was possible to work as a signal to trigger an essential modification of synaptic connections between the two networks to shift the working memory from memory maintenance to retrieval phases. Simulated results demonstrated the importance of neurotransmitters that were involved in coupling the DMN and WMN. Specifically, AMPA terms could guarantee necessary oscillation in maintenance phase (Fig. 5), switching on and off NMDA channel between TPN and TNN in several certain timepoints would diverse neural activities in the network model (Figs. 7, 8), and an abnormal NMDA conductance increase would interrupt a normal ongoing working memory maintenance phase (Fig. 9). For a complete working memory process, our simulated results demonstrated that the shift of working memory phases was primarily due to changes in excitatory neurotransmitter conductance between WMN and different DMN subnetworks (Fig. 11).

The conductance between two networks would profoundly affect the working memory function in some physiological experiments (Takei et al. 2016). For example, lower glutamate/GABA ratio could induce stronger DMN inhibition (Gu et al. 2019), while the increase of this ratio could induce a weakening DMN inhibition. Abnormal concentration ratio of inhibitory to excitatory neurotransmitters is a typical characterization of depressive psychiatric disorders (Kendell et al. 2005). A specific abnormal ratio of neurotransmitter concentrations might even cause the patient to recall a specific event as an intensification of memory, which was a possible cause of depression symptom (Figueroa et al. 2017). An excited DMN was still able to inhibit the WMN, but the overactivity of DMN might manifest itself in actual brain activity as a characteristic of cognitive disorders and psychiatric problems, like PTSD and MDD (Kaiser et al. 2019). Those patients might have difficulties in doing working memory tasks in actual behavioral experiments (Anticevic et al. 2012).

Previous studies have demonstrated that the TPN built on I–F neurons with synaptic gating mechanisms can work as the WMN independent of TNNs (Compte 2000; Wei et al. 2012). Our results further confirmed that when the TPN was coupled with the TNN, neurons in the TPN fired more pronouncedly and more intensely, so that memory contents in the TPN became more sustainable. According to the simulated results of sequentially presented stimuli (Fig. 10), only if the second stimulus immediately followed the first stimulus and had a longer presentation duration, the second stimulus had the potential to be remembered. This simulated mechanism could be used to interpret a macro-behavioral chunk theory of working memory.

The number of the sequential stimuli was possibly determined before the TPN–TNN system got into a steady state (Fig. 10) without adjusting any internal synaptic parameters. After the TPN–TNN system went into an energy-determined stable state, the TPN–TNN network was only able to memorize the determined sequential stimuli, and it was relatively hard to remember additional stimuli any more (Fig. 10d–f). The initial stimulus, representing an initial memorized chunk, was hard to be distracted. It behaviorally implicated that once a content A was stored in the networks as working memory, the networks continued keeping the content A until a change of neurotransmitter concentration that brought the working memory process to a halt (Fig. 9). However, this proposal contradicted results from the well-known WMN model consisting of one TPN (Compte 2000). In the WMN model with only one TPN, a transient strong external stimulus could stop working memory maintenance phase (Mayer et al. 2016). However, when the WMN model have a TPN coupled with a TNN, the present study found that the neurotransmitter dynamics rather than the external stimulus was more important to stop a maintained memory. Compared to simulated results with only one TPN (Compte 2000), maintained components in working memory were more difficult to be altered when there was a TNN coupled with a TPN. This implied that the inhibitory effect of the TNN as the DMN on the TPN could significantly reinforce the maintenance phase of working memory and make the presence of the memory content more robust.

Using the phase transformation method (Figs. 7,8) and energy analysis method (Eqs. 26–28), as well as the network model with coupled networks (Fig. 2), a theoretical simulation of the complete three phases of working memory was constructed in this article. For the reason that the WMN was connected to different brain regions in the DMN alternately in a complete working memory process, one single TPN with two TNNs were chosen for the network architecture. The connection weights within each of the two TNNs were fixed during the whole working memory process, only NMDA channels between the TPN and TNN1, between the TPN and TNN2 would be switched on or off in different memory processes. Our simulation results on three phases of working memory showed that the positive and negative correlations exhibited by the coupling of DMN and WMN could be integrated, and that their alternation appearance might be a plausible explanation for the working memory mechanism.

Such a simulation approach could help to explore the essential mechanism of working memory, but it had some following limitations. (1) The simulation process in this article ignored changes of synaptic connections within each network as well as the other types of synaptic connections between networks when working memory phases were shifted. (2) This method also did not perform nonlinear correlations (e.g., MIC) which would account more convincingly for the consistency of phase synchronization with or without antagonism in terms of correlation analysis. (3) Some physiological studies have shown an increase in γ-band activity of local field potentials in working memory experiments, but more experimental data still have shown that working memory is more correlated with theta-band (4–8 Hz) activity (Lee et al. 2005). The rhythmic activity of population neurons was in γ band (≈50 Hz) at the energy level simulated by kinetic equations in our research. If slower frequencies need to be simulated, it may be necessary to modify the network from the basic I–F model, which will inevitably involve a large number of parameter adjustments, and the I–F model with synaptic gating used in this study may not be an optimal choice. (4) Our conclusion and proposed model were suitable to researches related to coordination of the DMN and WMN at present. As the DMN includes a collection of several brain regions, different brain regions may play different roles in the DMN and these regions may together determine the global dynamics of the DMN. However, we have not clearly demonstrated how interactions between sub-regions in DMN significantly influence the DMN dynamics and its related functions. This could be an important question required for further investigation.

## Conclusion

Results in this study demonstrated the possibility of co-existence of positive and negative correlations between the DMN and WMN. Positive correlation was obtained when both the networks showed oscillatory activities with similar frequencies simultaneously, while negative correlation was obtained when one of the networks was suppressed and its rhythmic activity became slow waves or noisy signals. These interesting simulation results revealed that the DMN was coupled with the WMN in complex and divers ways. Our simulated results demonstrated that switch on or off synaptic channels between the WMN and sub-networks in the DMN could be possible mechanisms to explain transition of multiple activity patterns in the DMN–WMN network observed in the fMRI experiment (Piccoli, et al. 2015).

Overall, by further exploring the coupling mechanism between the WMN and DMN in the TPN–TNN model, this article identified how important the conduction of excitatory neurotransmitter NMDA between different networks was in a complete working memory process. We proposed a perspective that positive and negative correlations between DMN and WMN were not necessarily contradictory. The theoretical TPN–TNN model contributed to a better understanding the coupling mechanism between DMN and WMN and their functions in working memory. The existence of both negative and positive correlations between those two networks could help to explain the possible relationship between working memory impairment and abnormal DMN activity in pathology.

## Supplementary materials

The supplementary materials alongside this manuscript is parameter tables of the simulation codes. Parameter Index I pdf file contains constant parameters of synaptic connection in the simulation, basically similar to the parameters in theoretical model of Compte. A. (2000). σand J + in the “Parameters of excitatory interaction” table in index I could be change to achieve different results. Parameter Index II pdf file contains the neurotransmitter conductance (g) between each two neuronal populations. Conductance adjusted in this article has been marked as colored cells. Details could be seen in the notes under the parameter table. Simulation codes (Matlab R2014) could be obtained by e-mail the first author or the corresponding author, for it is not suitable to put codes in publishment.

## Supplementary Information

Below is the link to the electronic supplementary material.

Supplementary file1 (PDF 104 kb)

Supplementary file2 (PDF 138 kb)

## Data Availability

The underlying parameters, high-resolution figures and codes would be provided in the Supplementary Information files alongside the manuscript. The data is also available on request by e-mailing to corresponding author or first author.
