# Supplementary material for: Biophysical mechanism of the interaction between default mode network and working memory network
Source: Cogn Neurodyn. 2021 Apr 19;15(6):1101–24. doi: 10.1007/s11571-021-09674-1 (PMC8572310; doi:10.1007/s11571-021-09674-1)
Supplement: Supplementary file 1 — Supplementary file1 (PDF 104 kb) [file 11571_2021_9674_MOESM1_ESM.pdf]

Parameters of excitatory interaction

Parameter	Value	Annotation
n	2048	The number of pyramidal neurons in each network
Cm	0.5	Membrane capacitance（nF）
g_L	0.025	Leak conductance（mS）
V_L	-70	Resting potential（mV）
Vm_thre	-50	Threshold potential（mV）
Vm_reset	-60	Reset potential after an action potential（mV）
τ	2	Refractory period（ms）
f_ext	1	Frequency of external Poisson noise（kHz）
g_ext	0.00248	Synaptic conductance of external Poisson noise（mS）
σ	11.25	Gaussian parameter of selective weight in TPN & TNN2
J+	3.62	Gaussian parameter of selective weight in TPN & TNN2

Parameters of AMPA

Parameter	Value	Annotation
V_E	0	Reversal potential(mV)
τ_s	2	τ_{AMPA} in Eq.8 (ms)
α_s	1	Program substantial variable of δ in Eq.8

Parameters of NMDA

Parameter	Value	Annotation
V_E	0	Reversal potential(mV)
τ_s	100	$\tau_{NMDA,decay}$ in Eq.11 (ms)
α_s	0.5	β in Eq.11
C_Mg	1	Concentration of Mg^{2+}(mM)
τ_x	2	$\tau_{NMDA,rise}$ in Eq.10 (ms)
α_x	1	Program substantial variable of δ in Eq.10

Parameters of inhibitory interaction

Parameter	Value	Annotation
n	512	The number of interneurons in each network
Cm	0.2	Membrane capacitance（nF）
g_L	0.02	Leak conductance（mS）
V_L	-70	Resting potential（mV）
Vm_thre	-50	Threshold potential（mV）
Vm_reset	-60	Reset potential after an action potential（mV）
τ	1	Refractory period（ms）
f_ext	1	Frequency of external Poisson noise（kHz）
g_ext	0.0019	ynaptic conductance of external Poisson noise（mS）

Parameters of GABA

Parameter	Value	Annotation
V_I	-70	Reversal potential(mV)
τ_s	10	τ_{GABA} in Eq.9 (ms)
α_s	1	Program substantial variable of δ in Eq.9

Parameters of External Stimuli

Parameter	Value	Annotation
I_0	0.4	Maximum stimulation current(pA)
σ_s	2	Standard deviation of stimuli
