# Supplementary material for: Biophysical mechanism of the interaction between default mode network and working memory network
Source: Cogn Neurodyn. 2021 Apr 19;15(6):1101–24. doi: 10.1007/s11571-021-09674-1 (PMC8572310; doi:10.1007/s11571-021-09674-1)
Supplement: Supplementary file 2 — Supplementary file2 (PDF 138 kb) [file 11571_2021_9674_MOESM2_ESM.pdf]

Synaptic conductance (g) between pyramidal and interneuron population

Network (Brain region)	Neuronal signal transmission direction	NMDA	AMPA	GABA
Task Positive Network (dorsal lateral prefrontal cortex)	TPN pyramidal →TPN pyramidal	0.0001905	0.0001905*3.5/6.5	/
	TPN pyramidal →TPN interneuron	0.000146	0.0001460*3.5/6.5	/
	TPN interneuron →TPN pyramidal	/	/	0.0006681
	TPN interneuron →TPN interneuron	/	/	0.000512
Task Negative Network I (posterior cingulate cortex)	TNN I pyramidal →TNN I pyramidal	0.0001905*4	0.0001905*4*2.0/6.5	/
	TNN I pyramidal →TNN I interneuron	0.000146*4	0.000146*4*2.0/6.5	/
	TNN I interneuron →TNN I pyramidal	/	/	0.0006681*1.5
	TNN I interneuron →TNN I interneuron	/	/	0.000512*1.5
Task Negative Network II (inferior parietal lobule)	TNN II pyramidal →TNN II pyramidal	0.0001905*4	0.0001905*4*2.0/6.5	/
	TNN II pyramidal →TNN II interneuron	0.000146*4	0.000146*4*2.0/6.5	/
	TNN II interneuron →TNN II pyramidal	/	/	0.0006681*1.5
	TNN II interneuron →TNN II interneuron	/	/	0.000512*1.5
Interaction between TPN (DLPFC) & TNN I (PCC)	TPN pyramidal →TNN I pyramidal	k5/2048*0.0005	k5/2048*0.0005*3.5/6.5	/
	TPN pyramidal →TNN I interneuron	k5/2048*0.0005	k5/2048*0.0005*3.5/6.5	/
	TNN I pyramidal →TPN pyramidal	60/2048*0.0005	60/2048*0.0005*3.5/6.5	/
	TNN I pyramidal →TPN interneuron	60/2048*0.0005	60/2048*0.0005*3.5/6.5	/
Interaction between TPN (DLPFC) & TNN II (IPL)	TPN pyramidal →TNN II pyramidal	k5/2048*0.0005	k5/2048*0.0005*3.5/6.5	/
	TPN pyramidal →TNN II interneuron	k5/2048*0.0005	k5/2048*0.0005*3.5/6.5	/
	TNN II pyramidal →TPN pyramidal	60/2048*0.0005	60/2048*0.0005*3.5/6.5	/
	TNN II pyramidal →TPN interneuron	60/2048*0.0005	60/2048*0.0005*3.5/6.5	/
Interaction between TNN I (PCC) & TNN II (IPL)	TNN I pyramidal →TNN II pyramidal	0.0001905*2	0.0001905*4*2.0/6.5	/
	TNN I pyramidal →TNN II interneuron	0.0001460*2	0.0001460*4*2.0/6.5	/
	TNN II pyramidal →TNN I pyramidal	0.0001905*2	0.0001905*4*2.0/6.5	/
	TNN II pyramidal →TNN I interneuron	0.0001460*2	0.0001460*4*2.0/6.5	/

*Yellow cells could be adjusted depending on how strong the oscillation caused by AMPA is needed .

*According to the Compte. A.(2000), the AMPA/NMDA ratio equals to 3.5/6.5.

*Blue cells would be switched to 0 during the working memory encoding and retrieval process, while green cells would be 0 in maintenance process. Parameter k5 and gaussian parameters could be adjusted to change the inhibition level between WMN and DMN. .

*Parameters of synaptic conductance in 3.1-3.3 part were mainly consistent to values of TPN, TNN1, and interaction between TPN & TNN1 in this sheet.
